# Mining Grapevine Downy Mildew Susceptibility Genes: A Resource for Genomics-Based Breeding and Tailored Gene Editing

**DOI:** 10.3390/biom11020181

**Published:** 2021-01-28

**Authors:** Carlotta Pirrello, Tieme Zeilmaker, Luca Bianco, Lisa Giacomelli, Claudio Moser, Silvia Vezzulli

**Affiliations:** 1 Research and Innovation Centre, Edmund Mach Foundation, Via E. Mach 1, 38010 San Michele all’Adige, Italy; carlotta.pirrello@fmach.it (C.P.); luca.bianco@fmach.it (L.B.); giacomelli.scienzagrapes@gmail.com (L.G.); claudio.moser@fmach.it (C.M.); 2 Department of Agricultural, Food, Environmental and Animal Sciences, University of Udine, Via delle Scienze 206, 33100 Udine, Italy; 3 SciENZA Biotechnologies B.V., Sciencepark 904, 1098 XH Amsterdam, The Netherlands; T.Zeilmaker@enzazaden.nl

**Keywords:** disease resistance, DLO, DMR, next-gen amplicon sequencing, SNP, susceptibility genes, *Vitis* spp.

## Abstract

Several pathogens continuously threaten viticulture worldwide. Until now, the investigation on resistance loci has been the main trend to understand the interaction between grapevine and the mildew causal agents. Dominantly inherited gene-based resistance has shown to be race-specific in some cases, to confer partial immunity, and to be potentially overcome within a few years since its introgression. Recently, on the footprint of research conducted in Arabidopsis, putative genes associated with downy mildew susceptibility have been discovered also in the grapevine genome. In this work, we deep-sequenced four putative susceptibility genes—namely *VvDMR6.1*, *VvDMR6.2*, *VvDLO1*, *VvDLO2*—in 190 genetically diverse grapevine genotypes to discover new sources of broad-spectrum and recessively inherited resistance. Identified Single Nucleotide Polymorphisms were screened in a bottleneck analysis from the genetic sequence to their impact on protein structure. Fifty-five genotypes showed at least one impacting mutation in one or more of the scouted genes. Haplotypes were inferred for each gene and two of them at the *VvDMR6.2* gene were found significantly more represented in downy mildew resistant genotypes. The current results provide a resource for grapevine and plant genetics and could corroborate genomic-assisted breeding programs as well as tailored gene editing approaches for resistance to biotic stresses.

## 1. Introduction

The development of disease-resistant varieties is a convenient alternative to chemical control methods to protect crops from diseases. When it recognizes and invades plant tissues and a plant-pathogen interaction is established, the pathogen is faced with the host response, which involves the activation of signals that translate into a rapid defense response. This immune response helps the host plant to avoid further infection of the pathogen [1]. To suppress this immunity, pathogens produce effector molecules to alter host responses and support compatibility. In turn, plants evolved the ability to recognize these effectors by resistance (*R*) genes. The majority of *R* genes encode nucleotide-binding leucine-rich-repeat (NBS-LRR) proteins. Since *R* genes are specifically directed towards highly polymorphic effector molecules or their derivatives, this kind of immunity is dominantly inherited, mostly race-specific, and rapidly overcome by the capacity of the pathogen to mutate [2]. Analyses of whole-genome sequences have provided and will continue to provide new insights into the dynamics of *R* gene evolution [3].

Besides the established *R* gene model, the susceptibility (*S*) gene model was more recently defined. All plant genes that facilitate infection and support compatibility can be considered *S* genes [4]. They can be classified into the following three groups based on the point at which they act during infection: those involved in early pathogen establishment, those involved in modulation of host defenses, and those involved in pathogen sustenance [5]. The concept of susceptibility genes was first explored in barley by Jorgensen (1992) [6] with the *MLO* (Mildew resistance Locus O) gene involved in susceptibility to powdery mildew. Later, *mlo* mutants were identified also in cucumber, melon, pea, tomato, and tobacco [7]. Other analyzed susceptibility genes are the so called *DMR* (*Downy Mildew Resistant*) genes firstly characterized in Arabidopsis by Van Damme et al. (2005; 2008) [8,9], and *DLO* (*DMR-like Oxygenases*) [10]. *DMR6* and *DLO* are paralogs, their separation occurred prior to the appearance of flowering plants [11]. Both genes encode a 2-oxoglutarate (2OG)-Fe(II) oxygenase [9,10]. The putative functions of *DMR6* and *DLO* were defined by Zhang et al. (2013) and Zhang et al. (2017) [10,12]. DMR6 and DLO are involved in salicylic acid (SA) catabolism. More specifically, DMR6 functions as a SA-5-hydroxylase (S5H) whereas DLO functions as a S3H, converting the active molecule of SA into 2,5-DHBA (dihydrobenzenic acid), and 2,3-DHBA inactive forms, respectively [10,12]. Being involved in SA catabolism, *DMR6* and *DLOs* fall into the category of *S* genes acting in the negative regulation of immune signaling. Their inactivation could improve plant resistance. Initially the *Arabidopsis thaliana dmr6* mutant was isolated from a chemically mutagenized population for its resistance to *Hyaloperonospora arabidopsidis*, the downy mildew (DM) causal agent in this species [8]. Orthologues were readily identified in tomato [13] as well as many other crops [14,15] and fruit trees [11,16]. Mutations in *DMR6* confer broad-spectrum resistance; *Sldmr6-1* tomato mutant plants show resistance against *Phytophthora capsici*, *Pseudomonas siringae*, and *Xanthomonas* spp. [13].

In order to identify mutations and to deepen their impact on plant performance, studies of genetic diversity are essential and have been extensively performed in the plant kingdom, although compared to animals and humans their sequel is still in its infancy. A SNP (Single Nucleotide Polymorphism) provides the ultimate form of molecular marker, based on differences of individual nucleotide bases between DNA sequences [17]. SNPs are more abundant in the genome and more stably inherited than other genetic markers [18] and they can be classified into random, gene targeted, or functional markers according to their localization [19]. The discovery of functional SNPs—that cause phenotype variations—is challenging and scarcely described in the literature. In particular, functional SNPs were used to target flowering time and seed size in lentil [20], midrib color in sorghum [21], leaf hair number in turnip [16], grain length [22], and blast resistance in rice [23].

A variety of approaches have been adopted to identify novel SNPs [24]. In the last decade, computational approaches have dominated SNP discovery methods due to the advent of next-generation sequencing (NGS) [25], followed by third-generation sequencing platforms (TGS) [26], and the consequent ever-increasing sequence information in public databases. Since the first whole plant genome to be sequenced [27], de novo and reference-based SNP discovery and application are now feasible for numerous plant species. Large-scale SNP discovery was performed in almost all sequenced plant genomes such as maize [28], Arabidopsis [29], rice [30], rapeseed [31], potato [32], and pepper [33]. On the method side, Genotyping-By-Sequencing (GBS) has recently emerged as a promising genomic approach to explore plant genetic diversity on a genome-wide scale [34], followed by the more cost-effective Genotyping-in-Thousands by sequencing (GT-seq) [35]. Genetic applications such as linkage mapping, phylogenetics, population structure, association studies, map-based cloning, marker-assisted plant breeding, and functional genomics continue to be enabled by access to large collections of SNPs [36]. In parallel to SNP discovery based on whole genome sequencing, amplicon sequencing has also been successfully applied in plants [37,38,39,40] although less frequently than in bacteria [41] or viruses [42].

Recently, as advocated by Gupta et al. (2001) [43], progress has also been made in the development and use of SNPs in woody plants, including some crop and tree species as apple [44], walnut [45], sweet cherry [46], pear [47], coffee [48], and grapevine [49,50]. This phenomenon is due to the boost in the sequencing of cultivated plant genomes to provide high-density molecular markers for breeding programs aimed to crop improvement as well as to elucidate evolutionary mechanisms through comparative genomics [51,52]. In grapevine a great deal of progress has been made from the first SNP identification in the pre-genomic-era [53] to the sequencing of the whole genome of several *Vitis vinifera* cultivars [54,55,56,57,58,59], to the very recent report of the genome sequence of *Vitis riparia* [60] and the diploid chromosome-scale assembly of *Muscadinia rotundifolia* [61]. The last two studies represent a turning point on the scavenging of genomes that are donors of disease resistance traits. This issue in *Vitis* spp. is tackled by identifying *R* loci, underlying *R* genes, through quantitative trait loci (QTL) analysis in different genetic backgrounds. Nowadays, 13 *R* loci against powdery mildew and 31 to DM have been identified with different origins, mainly from American and Asian wild species [62,63].

A promising approach to cope with disease resistance is represented by the study of *S* loci. Based on a high-resolution map, Barba et al. (2014) [64] identified on chromosome 9 a locus (*Sen1*) for powdery mildew susceptibility from ‘Chardonnay’, finding evidence for quantitative variation. Moreover, on the footprint of research conducted on model plants, genes associated with mildew susceptibility have been discovered and dissected also in the grapevine genome. 17 *VvMLO* genes, orthologues of the Arabidopsis *MLOs*, were identified and a few members showed transcriptional induction upon fungal inoculation [65,66]. Lately, more insights in terms of powdery mildew resistance has been achieved by silencing of four *VvMLO* genes through RNAi in grapevine [67].

In this research, we aim to investigate the diversity of the *DMR6* and *DLO* genes in a wide set of *Vitis* spp. to broaden our knowledge about the genetic variation present and about the impact on the protein structure and function. This information will represent a resource to enhance our knowledge of possible alternative or integrative solutions, as compared to the use of *R* loci to be applied in plant molecular breeding strategies.

## 2. Materials and Methods

### 2.1. Genetic Material and Target Genes

In the current study, the four *VvDMR6.1*, *VvDMR6.2, VvDLO1*, and *VvDLO2* genes were scouted in 190 grapevine genotypes (Table 1, Table S1).

Out of these, 139 (73%) are *Vitis* hybrids, 28 (15%) are *V. vinifera* varieties, 12 (6%) belong to wild *Vitis* species and additional 11 (6%) are ascribed as hybrids/wild species. Phenotypic data about DM resistance degrees were retrieved from literature, public databases, and unpublished information. Pairwise alignment [68] was performed in order to define nucleotide identity between investigated genes.

### 2.2. Amplicon Sequencing and Read Processing

Genomic DNA was extracted from young grapevine leaves using DNeasy Plant Mini Kit (QIAGEN, Hilden, Germany), according to the manufacturer’s protocol, and then used to produce amplicons for deep sequencing. PCR on the templates was performed using Phusion High-Fidelity Polymerase (NEB, Ipswich, MA, USA) according to the manufacturer’s protocol. Primers were specifically designed to amplify 250 bp of the coding regions of target genes and barcoded followed by in-house sequencing using the Illumina MiSeq platform (Table 1). A total of 19 amplicons was sequenced including six amplicons for *VvDMR6.1*, seven amplicons for *VvDMR6.2*, four amplicons for *VvDLO1*, and two amplicons for *VvDLO2*. Obtained amplicons were then mapped on the PN40024 12X reference genome [54] considering the latest V2 gene prediction [69,70] through Burrows–Wheeler alignment (BWA) [71] with no filter on mapping quality.

### 2.3. Sanger Sequencing

Thirteen impacting mutations (six in *VvDMR6.1*, two in *VvDMR6.2*, two in *VvDLO1*, three in *VvDLO2*) in 17 genotypes (12 hybrids, one *V. vinifera*, two wild species, two hybrids/wild species) in 25 combinations (Table S2) were chosen according to their representativeness of the overall results and to the availability of plants in situ. Previously extracted DNA was used to produce 12 targeted Sanger amplicons (six in *VvDMR6.1*, two in *VvDMR6.2*, two in *VvDLO1*, two in *VvDLO2*) by PCR using Phusion High-Fidelity DNA Polymerase (Thermo scientific) according to the manufacturer’s protocol. Purification was made enzymatically with ExoSAP-IT PCR Product Cleanup Reagent (Applied Biosystems Inc., Foster City, CA, USA) according to the manufacturer’s instructions. 3.2 μM of forward or reverse primer were then added to the sample and sequencing was performed using the BigDye Terminator Cycle Sequencing Ready Reaction Kit v3.1 (Applied Biosystems Inc.) in ten μL final volume. Sequencing reactions were performed using a 2 min initial denaturation step, followed by 25 cycles at 96 °C for 10 s, 50 °C for 5 s, and 60 °C for 4 min and then purified from unincorporated primer and BigDye excess through Multiscreen384SEQ Sequencing reaction Cleanup Plate (Millipore, Carrigtwohill, Co. Cork, Ireland). Capillary electrophoresis of the purified products was performed on a 3730 × l DNA Analyzer (Applied Biosystems Inc.). Pregap4/Gap4 from Staden Package software package [72] were used to align DNA sequence electropherograms and scan all polymorphic sites.

### 2.4. Data Mining and Protein Model

Variant calling was performed by BCFtools [73] using the following settings: minimum mapping quality 20; minimum genotype quality 20; minimum base quality 20; maximum per sample depth of coverage 1000; minimum depth of coverage per site 10; keep read pairs with unexpected insert sizes (for amplicon sequencing). Filtering of results was done with VCFtools [74] to exclude all genotypes with quality below 20 and include only genotypes with read depth ≥10.

SnpEff toolbox was used to further discriminate variants according to their impact (MODIFIER, LOW, MODERATE or HIGH accordingly to the user’s manual) on gene sequence [75]. Elected-impacting variants were then subject to SIFT (sorting intolerant from tolerant) [76] analysis to assess the tolerance of amino acid variants on the protein primary structure, based on the alignment with sequences in SWISS-PROT/TrEMBL database. Only not tolerated mutations were considered for a last impact evaluation based on variants chemical-physical properties according to Betts and Russel (2003) [77]. Both SnpEff and SIFT algorithms were used with default parameters settings.

Data obtained from mapping and variant calling were dissected to extrapolate overall genetic information on the studied genotypes. Amplicons were classified according to their level of polymorphism. All the other parameters were calculated considering all genotypes and the various taxon. For each gene, frequencies of occurring mutation arrangement were calculated along with mutation frequency, triallelic variants occurrence, and MAF. PHASE v2.1 software [78] was used for haplotype reconstruction and frequency calculation using PN40024 as the reference genome [54]. The genotypes belonging to specific classes (carried haplotypes) were linked in contingency tables to the phenotypic trait according to OIV 452(-1) [79]. Pearson’s Chi-squared Tests for Count Data were performed on each locus separately.

Sequences of bonafide (*) and putative DMR6 and DLO orthologues were collected from literature [11,13,14,80] and available databases (Plaza 3.0) [81] and aligned using ClustalW (https://www.ebi.ac.uk/Tools/msa/clustalo/).

Genes carrying mutations confirmed by Sanger sequencing were subjected to a homology detection and three-dimensional structure prediction using the HHpred tool of MPI Bioinformatic Tools [82] available at https://toolkit.tuebingen.mpg.de/#/tools/hhpred. The algorithm found a Thebaine 6-O-demethylase [83] as the protein sequence with three-dimensional structure available (PDB coordinates: 509W) and highest homology to VvDMR6 and VvDLO and it produced a three-dimensional model carrying the mutations using the MODELLER software [82]. The three-dimensional structure was visualized to better understand the impact of the mutations on the wild type protein structure.

## 3. Results

### 3.1. Sequencing and Mapping

*VvDMR6.1* and *VvDMR6.2* shared 46.7% nucleotide identity, *VvDMR6.1* and *VvDLO1* 44.8%, *VvDLO1* and *VvDLO2* 38.9%, all other comparisons resulted in a nucleotide identity lower than 40%. In order to identify potentially disrupting mutations, coding sequences of the *VvDMR6.1*, *VvDMR6.2, VvDLO1*, and *VvDLO2* genes (Table 1) from 190 genotypes (Table S1) were deep-sequenced and mapped on the reference genome PN40024 12X V2 (see Section 2). In total, 12,476,502 reads were produced. *VvDMR6.1* was covered by 5,450,614 reads (44%), *VvDMR6.2* by 3,476,587 (28%)*, VvDLO1* by 3,270,318 (26%), and *VvDLO2* by 278,983 (2%). The highest coverage was detected in hybrids with a total of 9,357,649 reads (75%), followed by *vinifera* with 1,333,887 (11%), hybrids/wild species with 964,847 (8%) and wild species with 814,225 (6%).

**Figure 1:**
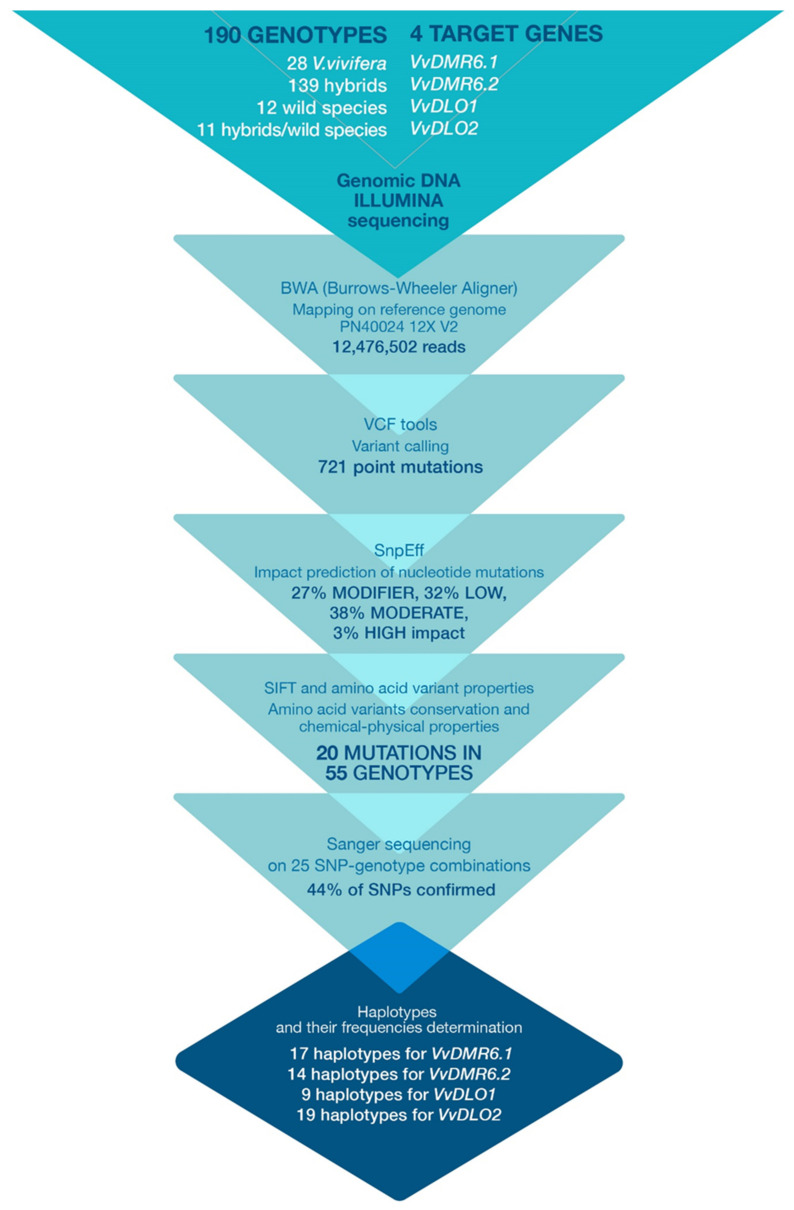
Flow chart of the analysis—tools and criteria—of sequencing data and results obtained downstream of each step.

A total of 738 mutations were detected by comparing the aligned reads to the Pinot Noir reference genome; 17 (~2%) short In/Dels and 721 point mutations, including heterozygous (56%) and homozygous (44%) SNPs (Figure 1).

### 3.2. Genetic Diversity Assessment

Amplicons were classified according to their rate of polymorphism: from the most polymorphic VvDLO2_1 (~13% of the total mutations); to the ones carrying ~8% of mutations VvDMR6.1_3, VvDMR6.1_2, VvDMR6.2_3 gradually decreasing to the lowest rate of polymorphism (less than 3%) in VvDMR6.2_7 and VvDLO1_4. Moreover, out of a total 738 mutations, 25 (~3.4%) triallelic variants were detected of which 13 in hybrids, eight in wild species, nine in *vinifera* varieties and eight in hybrid/wild species. Triallelic mutations were mainly found in *VvDLO2* (~1.6%) followed by *VvDMR6.1* (~1%), *VvDMR6.2* (~0.4%), and *VvDLO1*.

Considering the 696 biallelic mutations in all genotypes, 75% were transitions (A↔G, C↔T) and 25% were transversions (A↔C, A↔T, C↔G, G↔T) with a transition/transversion ratio of three. Both *vinifera* varieties and hybrids show the same assortment with 77% transitions and 23% transversions. In wild species the percentages were 73% and 27% respectively, while 71% and 29% were the values observed in hybrid/wild species.

SNP frequency was calculated both as average across all genes as well as per gene for every taxon. *Vinifera* varieties showed the lowest average frequency (~15 SNPs per Kb) with high differences between the target genes: ~33 SNPs per Kb in *VvDMR6.1*, ~22 SNPs per Kb in *VvDMR6.2*, ~18 SNPs per Kb in *VvDLO1*, and ~7 per Kb in *VvDLO2*. Moreover, the detected average frequency was ~18 SNPs per Kb in both wild species and hybrid/wild species, while they showed respectively ~23 per Kb and ~39 per Kb in *VvDMR6.1* ~20 and ~17.8 SNPs per Kb in *VvDMR6.2*, ~13 and 11 SNPs per Kb in *VvDLO1* and, ~22 and 20 SNPs per Kb in *VvDLO2*. Hybrids showed a higher average frequency (~28 per Kb) due to the dramatically high frequency values in *VvDMR6.1* (~75 per Kb) and in *VvDMR6.2* (~50 per Kb), ~38 SNPs per Kb in *VvDLO1* and 11 per Kb in *VvDLO2*.

In the current work, minor allele frequency (MAF) was calculated for each biallelic mutation. MAF values 0.01 ≤ *x* ≤ 0.05 were represented by the 29% of mutations detected in all genotypes, in particular the 23%, 0%, 2%, and 3% in hybrids, wild species, *vinifera* varieties and hybrids/wild species, respectively. MAF values 0.05 < *x* ≤ 0.1 were represented by 3% of the mutations in all genotypes as well as in wild species and by 2% in hybrids, *vinifera* varieties and hybrid/wild species. 0.1 < *x* ≤ 0.3 MAF values were represented by the 5% of mutations in all genotypes as in hybrids; wild species and *vinifera* varieties represented them by the 4% of their mutations and hybrid/wild species by the 2%. A very low percentage of mutations showed MAF 0.3 < *x* ≤ 0.5: 3% for all genotypes, hybrids and *vinifera*; 2% for wild species and hybrid/wild species.

### 3.3. Mutation Impact Evaluation

In the current study, upon the variant discrimination performed according to their impact on codon sequence, 27% of total mutations (in particular, 27% in *VvDMR6.1*, 25% in *VvDMR6.2*, 30% in *VvDLO1* and 25% in *VvDLO2*) were classified as “MODIFIER”: falling into intronic regions or upstream/downstream the gene. “LOW” impact variants, responsible for synonymous mutations or falling into splice regions, represented the 32% of the total mutations: 36% in *VvDMR6.1*, 32% in *VvDMR6.2*, 32% in *VvDLO1*, and 28% in *VvDLO2*. Of the total mutations, 38% (in particular, 35% in *VvDMR6.1*, 40% in *VvDMR6.2*, 35% in *VvDLO1* and 43% in *VvDLO2*) were non-synonymous variants and therefore classified with “MODERATE” impact. These percentages are partially confirmed in *vinifera* by Amrine et al. (2015) [84], with ~90% of MODIFIER and LOW mutations and ~8% non-synonymous variants in gene sequence. The lowest number of variants (in average 3%: 2% in *VvDMR6.1*, 2% in *VvDMR6.2*, 3% in *VvDLO1* and 4% in *VvDLO2*) was classified with “HIGH” impact as being responsible for sequence frameshifts or premature stop codons. Following the filtering of mutations classified as “MODERATE” and “HIGH” (41%) in order to discriminate amino acid variants according to their conservation, these variants were further checked and mutants carrying different chemical/physical properties from the reference were chosen. Finally, results from both analyses on amino acid sequence were cross-referenced and 20 mutations were elected as potentially affecting the protein structure: 6 in VvDMR6.1, 4 in VvDMR6.2, 4 in VvDLO1, and 6 in VvDLO2 (Table S3, Figure 1).

Twenty-five genotype-SNP combinations were selected for confirmation via Sanger sequencing. 44% of the mutations were confirmed by Sanger sequencing, while 56% were not, indicating a certain discrepancy from Illumina sequencing results. In *VvDMR6.1*, two mutations out of six polymorphisms were validated in one genotype each. The same variant in *VvDMR6.2* was confirmed in three individuals. In *VvDLO1* the confirmed variants were two, both in two different genotypes. Two individuals shared only one mutation in *VvDLO2*. Validated variants spanned among all the scouted genes, and the distribution of genotypes carrying confirmed mutations fairly represented the starting taxon assortment (six hybrids, one wild species, two hybrid/wild species individuals). For each gene, there were mutations that were both confirmed and unconfirmed depending on the genotype, and some individuals carried both confirmed and unconfirmed variants in the same gene. We classified Sanger-investigated variants according to their read coverage (DP) and to their genotype quality (GQ). Out of the total 25 variants taken into account, 15 showed DP < 100 and 10 mutations with DP > 100 of which only one with DP close to 1000. While within 15 mutations with 10 < DP < 73 only four NGS results (27%) were confirmed, 7 out of the 10 variants (70%) with DP > 100 could be confirmed via Sanger sequencing. Furthermore, seven variants out of 25 (28%) showed a GQ lower than 99, of these only two were confirmed by Sanger sequencing. The remaining 18 mutations (72%) had GQ = 99 and half (nine) of them were confirmed. Considering both DP and GQ values together, six out of the seven variants with GQ < 99 showed DP < 100 but still two of them were Sanger sequencing confirmed. While five out of the nine remaining confirmed mutations showed GQ > 99 and DP > 100, two variants were with 50 < DP < 100. Of all the 20 impacting mutations considered (Table S3), only five were located at less than 60 nucleotides from amplicon or contig edge, and only one at less than 10 nucleotides. All the variants located on boundaries showed DP < 100; 50% of these edge mutations showed GQ < 99 and the other half GQ > 99. All the Sanger-confirmed variants were located far from amplicon ends, while only one was located on a reverse primer.

In order to provide robust results, only the validated mutations, corresponding to 11 genotype-SNP combinations, were selected for haplotype reconstruction and following analyses (Figure 1).

### 3.4. Mutated DMR and DLO Gene Combinations

Of the 190 studied genotypes, 55 showed at least one of the elected mutations: 37 hybrids, three *vinifera* varieties, six wild species and nine hybrid/wild species. 73% of individuals showed mutations only in one gene: 13% in *VvDMR6.1*, 29% in *VvDMR6.2*, 7% in *VvDLO1* and 24% in *VvDLO2*, while 26% were double mutants within six gene combinations and one genotype was mutant in three genes (Table S4). Haplotypes and their frequencies were determined for *VvDMR6.1*, *VvDMR6.2*, *VvDLO1*, and *VvDLO2* genes. Individuals carrying one impacting mutation per each gene were selected and the gene haplotypes were inferred taking into account all the flanking mutations showing at least MODERATE impact on the gene sequence (Table 2, Table S5). For *VvDMR6.1*, based on 14 SNPs, 17 haplotypes were calculated in 11 genotypes. The reference haplotype was the prominent (18.2% of frequency), all the others were unique, except for two haplotypes respectively shared by two individuals. No particular association between taxon and haplotype occurrence was observed. Regarding *VvDMR6.2*, 14 haplotypes were reconstructed based on 14 SNPs in 27 genotypes. The most shared haplotype (40.7%), showing two impacting mutations, was present in 12 individuals belonging to hybrids and, mainly in homozygous state, to *Vitis spp.*/hybrid individuals. The reference haplotype was the second one mostly represented, and then the third one showed 13% of frequency being shared by six hybrid genotypes. *VvDLO1* showed nine haplotypes based on 11 SNPs in 10 individuals. Besides the most recurrent reference haplotype (30%), the one with 20% of frequency encompassed two impacting mutations in one hybrid and two wild species. Sixteen SNPs in 25 genotypes were taken into account for *VvDLO2*, resulting in 19 haplotypes. Most haplotypes were unique or slightly shared, except for the reference one (34% of frequency) and two other main haplotypes (12% each) respectively shared by only and both hybrids and wild species (Table S5).

Integrating genotypic (haplotypic) data and available phenotypic OIV 452(-1) scores (Table 2), a chi-squared test was performed in order to check that genotypes belonging to specific classes (carried haplotypes) significantly led to the DM resistance trait. Interestingly, in *VvDMR6.2*, significance levels *p* = 0.0025 and *p* = 0.018 were respectively observed for haplotype number 10 and 8.

### 3.5. Mutation Mapping on Amino Acid Sequences and Protein Structural Model

**Figure 2:**
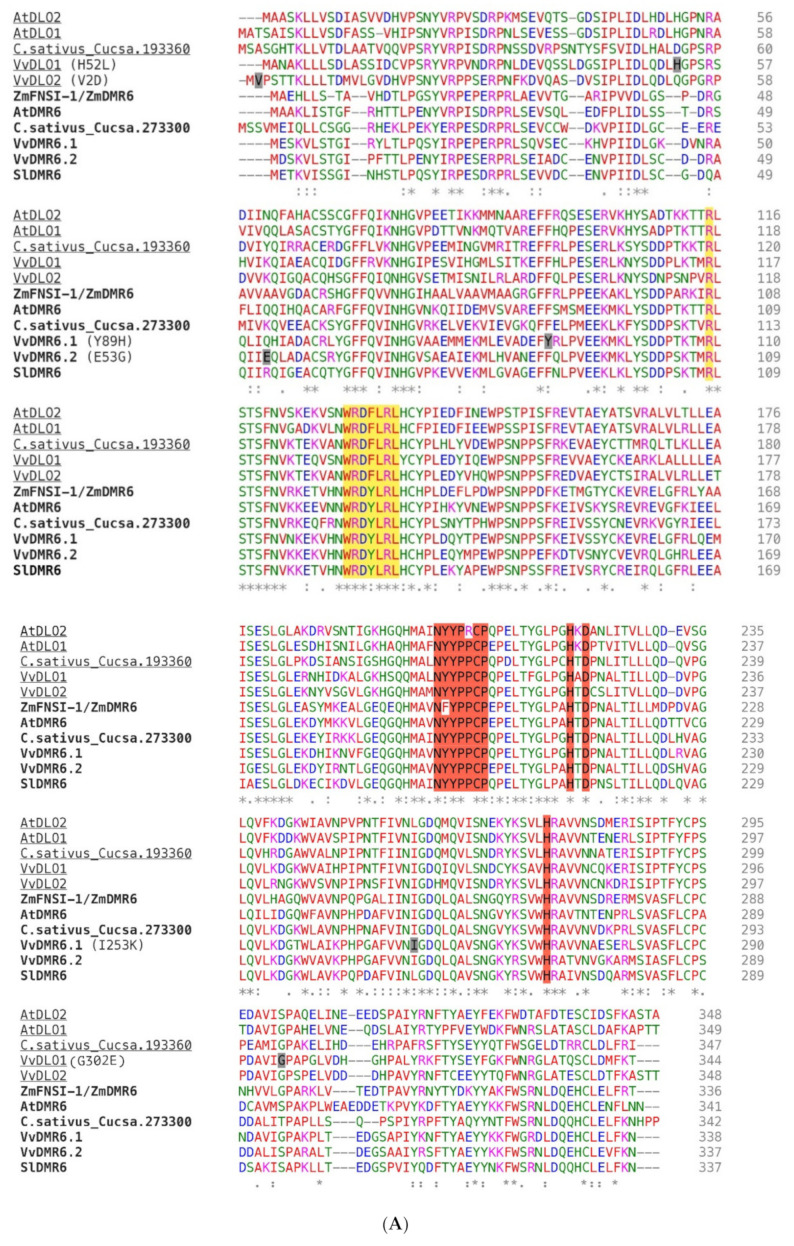

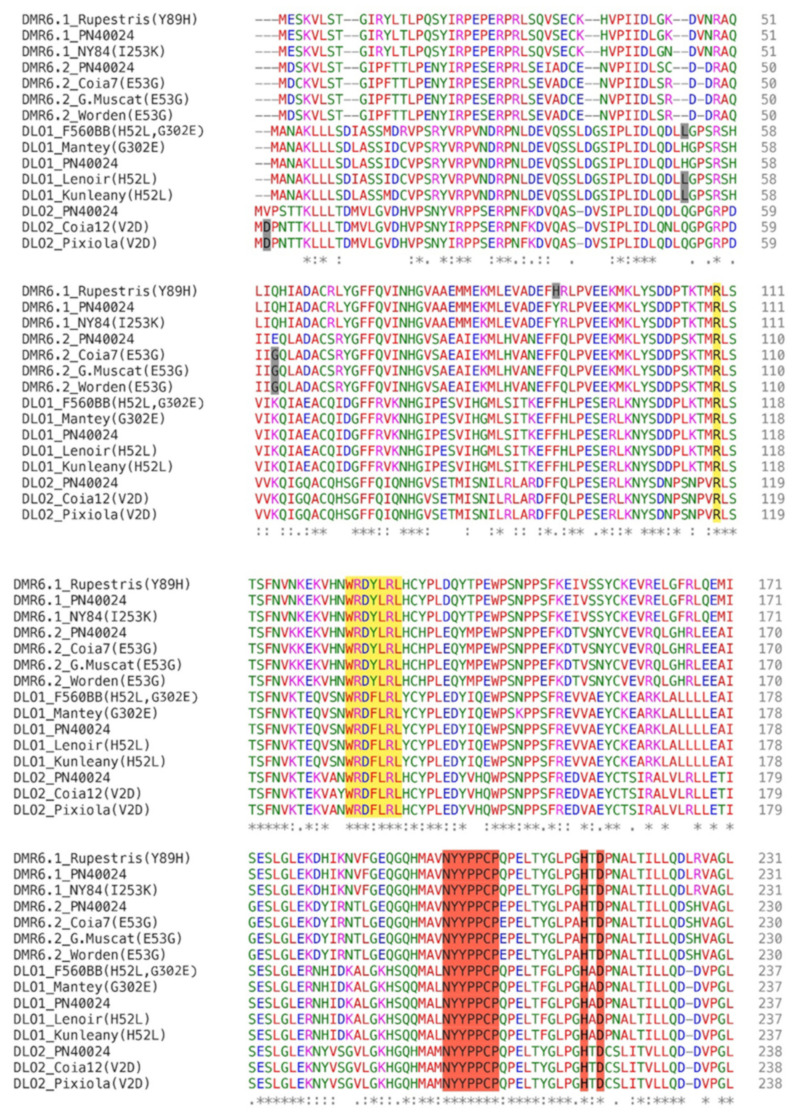

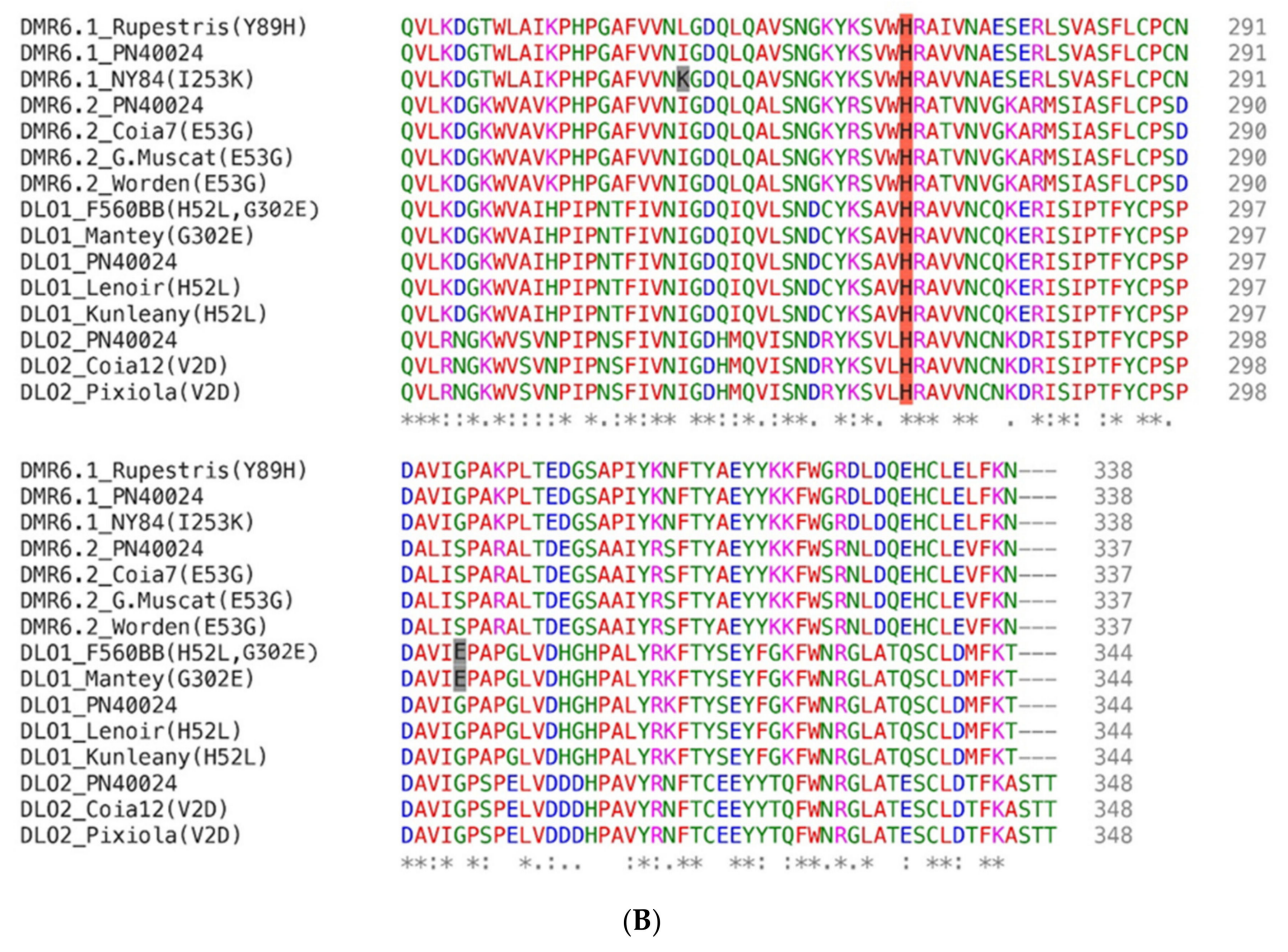
Amino acid sequence alignments. Amino acids important for the 2-DOG oxidase function (e.g., the NYYPPCP stretch responsible for binding the 2-oxoglutarate substrate and the iron-binding HDH triplet) are highlighted in red. The DLO-DMR6 characterizing motif WRDY/FLRL is highlighted in yellow; R124 within the WRDY/FLRL motif, and R108 of the Arabidopsis thaliana DMR6-1 sequence were shown to be essential for the function and are as well highlighted in yellow [80]. Amino acids that are changing in the different grapevine variants are indicated within parenthesis and their position is highlighted in grey on the sequence. (**A**) CLUSTALW alignment of bonafide DMR6 (in bold) and DLO (underlined) proteins from different species. C.sativus_Cucsa.193360 and C.sativus_Cucsa.273300 were identified as AtDMR6 orthologues in *Cucumis sativus* by Schouten at al. (2014) [14], although no experimental proof is provided. Bonafide DMR and DLO proteins are: *Zea mays* ZmFNSI-1/ZmDMR6, *A. thaliana* AtDMR6, AtDLO1, and AtDLO2; *Solanum lycopersicon* SlDMR6. The grapevine DMR6 and DLO proteins (VvDMR6.1, VvDMR6.2, VvDLO1, and VvDLO2) are those of the PN40024 reference genome. (**B**) CLUSTALW alignment of translated grapevine sequences. Abbreviations: Rupestris: *V. rupestris* du Lot, PN40024: *Pinot noir*-derived near-homozygous line, NY84: NY84.0100.05, F560BB: F560 Big Brown, G.Muscat: Golden Muscat. * (asterisk) indicates positions which have a single, fully conserved residue. : (colon) indicates conservation between groups of strongly similar properties - scoring > 0.5 in the Gonnet PAM 250 matrix. . (period) indicates conservation between groups of weakly similar properties - scoring =< 0.5 in the Gonnet PAM 250 matrix.

**Figure 3:**
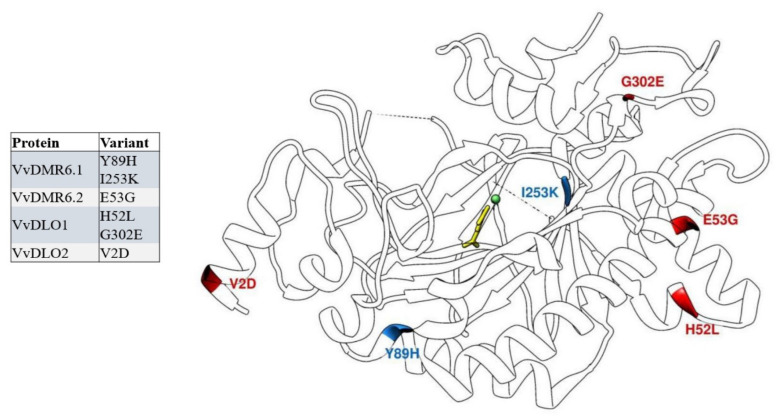
Protein structure model with detected impacting variants. In blue are residues located inside the protein while in red are those more exposed on the surface.

The amino acid variants corresponding to the mutations confirmed by Sanger sequencing were further investigated: (i) to estimate their conservation at the primary sequence level both within *Vitis* as well as in a larger group comprising other plant species (Figure 2A,B, Figure S1), and (ii) to evaluate their impact on the protein tertiary structure model (Figure 3).

Due the high sequence identity among them, the same protein three-dimensional model was used for mapping the mutations of all four proteins. Of the six amino acid substitutions two were found in VvDMR6.1 and VvDLO1 respectively, and one in VvDMR6.2 and VvDLO2 (Figure 3). All these mutations were non-conservative and therefore could potentially determine deep structural changes affecting also on the protein function. As depicted in Figure 3, four mutations appeared to be more exposed to the solvent, while the other two were buried inside the hydrophobic core of the proteins. Changes in the exposed amino acids are often less detrimental on the protein structure/function and this is the case of the V2D and H52L mutations. Although these mutations replaced a hydrophobic residue with a negatively charged one (V > D) and vice versa (H > L), being solvent exposed they do not seem of high impact on the protein structure. G302E and E53G mutations affect both steric hindrance and charge of the amino acid: glycine bearing the smallest side chain and glutamic acid bearing a bulky and negatively charged side chain. Also, for these two mutations, the location at the protein surface suggests that they may be tolerated and likely do not affect heavily protein function. The remaining mutations Y89H and I253K might instead have a much greater impact on the structure and function of VvDMR6.1, the sequence where they have been found. In this case, amino acids with hydrophobic character (Y and I) and positioned within the hydrophobic core of the globular protein are changed into positively charged amino acids (H and K).

## 4. Discussion

### 4.1. Wealth of Genetic Variability

The current survey revealed a high representation of triallelic mutations within our genotype panel, due to the great genetic variability considered. Analogously, the occurrence of triallelism is consistent with previous work in grapevine [86,87,88]. However, as reported by Bianco et al. (2016) [44] and Marrano et al. (2019) [45], triallelic variants are usually discarded in large scale SNP-based analyses for cost reasons (i.e., they require multiple probes in SNP arrays) and not necessarily because they are less accurate. The obtained results in terms of transitions/transversions slightly diverge from the usual ratio found in grapevine (~1.5 in Salmaso et al., 2004; Lijavetzky et al., 2007; Vezzulli et al., 2008; Vezzulli et al., 2008; ~2 Marrano et al., 2017) [86,87,88,89,90] as well as in beetroot [91], potato [92] and cotton [93], while they are much higher than in soybean [94] and almond [95].

Regarding the detected average of ~15 SNPs per Kb in *vinifera* genotypes, a comparable polymorphism rate (~14.5 SNPs per Kb in coding regions) was found in both cultivated (spp. *sativa*) and non-cultivated (spp. *sylvestris*) *vinifera* species by Lijavetzky et al. (2007) [86]. In contrast, Vezzulli et al. (2008) [87], estimated ~8.5 SNPs per Kb in cultivated *vinifera* and ~6 per Kb in wild *vinifera* individuals coding sequence. Moreover, studying different *Vitis* spp. genotypes, Salmaso et al. (2004) [89] observed an average of ~12 SNPs per Kb in the coding sequence of a set of genes encoding proteins related to sugar metabolism, cell signaling, anthocyanin metabolism, and defense. Based on the first Pinot noir consensus genome sequence, the average SNP frequency was estimated at four SNPs every Kb [55], compatible with the use of such molecular markers for the construction of genetic maps in grapevine [96]. Different polymorphism rates were found in other highly heterozygous tree species as peach (less than two SNPs per Kb) [97], black cottonwood (~3 per Kb) [98], almond (~9 per Kb) [95], and Tasmanian blue gum tree (~22 per Kb) [99], but all these results have to be carefully taken into account since different SNP calling methods can distort the comparison.

SNP informativeness depends on their reliability among individuals and species and their high transferability rates probably are not consistent with a direct impact on the genetic sequence (when in coding regions). Considering previous studies in grapevine, a larger representativeness of MAF values <0.1 was found in non-*vinifera* genotypes and rootstocks, non-cultivated *vinifera* showed a MAF 0.05 < *x* < 0.3 while MAF > 0.1 were severely represented by *vinifera sativa* [86,87,90,100]. As explained by Jones et al. (2007) [101] and Grattapaglia et al. (2011) [102], genotyping studies take advantage of different molecular markers, mostly relying on their informativeness. In this framework, SNPs are informative markers, and this peculiarity is calculated as MAF. SNPs are considered interesting for many goals when MAF values are >0.05 [103,104], but their main usefulness is due to the transferability across genotypes (>0.1) [86]. In the current study, the aim to focus on impacting mutations was achieved, since MAF ≤ 0.05 is a distinguishing mark for rare SNPs which affect the gene sequence and most likely the protein activity.

### 4.2. Relevance of Mutation Impact

In crops like tomato [105] and *Cucurbita* spp. [106], coding regions and whole genome sequence were scouted to find impacting mutations. A non-synonymous/synonymous mutation ratio of ~1.5 was found in tomato cultivars. In *Cucurbita* spp., the ratio was ~0.8 but only 9% of genetic variants showed HIGH or MODERATE impact in full genomic sequence, suggesting a great presence of intergenic mutations. In the walnut tree genomic sequence, Marrano et al. (2019) [45] identified 2.8% potentially impacting variants, while in the pear genome 55% of mutations were classified as missense and 1% with HIGH impact [107]. In grapevine, a significantly lower presence (0.7%) of HIGH impacting variants was observed in Thompson Seedless cultivar [108] compared to average percentages we observed in all taxa. The present aim to detect potentially disrupting mutations finds support in the great frequency of HIGH- and MODERATE-impact variants compared to the aforementioned research works on grapevine. Particular interest in the current results is given by the occurrence of impacting elected mutations in each one of the four scouted genes. Given the predicted compensative functional role of AtDMR6 and AtDLO in SA catabolism [10,12], obtained data may allow the use of *VvDMR6* and *VvDLO* genes in different combinations to enhance the impact of such homozygous mutations and likely avoid complementary effects.

Regarding the confirmation via Sanger sequencing, a borrowed attempt from clinical studies was tried herein on the overall grapevine Illumina sequencing results. In clinical research, reliability of variant calls is a fundamental precondition that requires the use of Sanger sequencing as gold standard to confirm NGS results and avoid false positives [109,110,111]. Incidentally, in order to avoid expensive and time-consuming extra analysis, some studies tried to set conditions according to which NGS-based variant calls can be considered definitive [112,113]. Although given the low number of tested samples we cannot draw a definitive conclusion that there is a direct correlation between these conditions and the reliability of Illumina sequencing-based calls, we observed that the most Sanger-confirmed variants (64%) showed DP > 100 and GQ = 99, while all ones were located away from the edges of the amplicons. The latter is in accord to Satya & DiCarlo (2014) [114], who report that variant calling accuracy decreases when SNPs are next to amplicon boundaries.

At this point, it is important to highlight the genetic complexity (high heterozygosity) of the studied genotype panel, which can unpredictably affect the Illumina probe as well as the Sanger sequencing primer annealing. Therefore, in order to provide reliable results, only validated mutations were selected for haplotype reconstruction and subsequent analyzes.

### 4.3. The Value of Haplotype Consideration

The reported broad genetic survey went back to the haplotype level. In three scouted genes out of four, the prominent haplotype belongs to the reference genotype (PN40024) which is a near-homozygous line [54] derived from the founder *vinifera* variety Pinot (noir) [115]. It is believed that the ancestral haplotype of a gene is the one showing the highest frequency while the rarest ones are the ones showing the most recent mutations occurring on the most shared haplotype [116], this hypothesis is supported by the fact that haplotype frequency is directly related to its age [117,118]. As advocated by Riahi et al. (2013) [119], domestication, hybridization with wild relatives and somatic mutations induced by vegetative propagation are the main reasons for the onset of genetic diversity between and among grapevine taxons.

Considering haplotypic data and available phenotypic OIV 452(-1) scores, two *VvDMR6.2* mutant haplotypes (number 10 and 8) were found more represented in DM resistant genotypes. It is relevant to highlight that none of the scouted target genes are underlying known resistance QTLs and no *R* loci discovered in grapevine so far were detected in the eight genotypes carrying these two haplotypes, except for the partial resistant *Rpv3-3* in three genotypes (Vezzulli S., personal communication). These observations suggest a potential effect of the mutant haplotypes in the defense response to DM. In grapevine, in addition to pursue association studies in large sample panels [120,121], some research works have lately been focusing on the haplotype investigation to dissect the relation between genetic diversity and cis-regulated gene expression in disease-related genes [122,123].

### 4.4. Scouting of Amino Acid Changes

DMR6 was identified as a putative 2-oxoglutarate (2OG)-Fe(II) oxygenase [9] and it revealed to share the WRD(F/Y)LR motif with DLO in flowering plant species [80]. Interestingly, Zeilmaker et al. (2015) [11] observed that non-conservative mutations in the catalytic sites (H212, H269, D214) of this protein were not able to restore susceptibility in an *Atdmr6.1* mutant background, in a complementation experiment. Unfortunately, no impacting mutation has been observed in any of these positions, but others have been identified that could potentially alter the structure of the protein. In particular, six mutations classified as impacting ones and confirmed by Sanger sequencing were further investigated by mapping on a three-dimensional model of the proteins and by analyzing the amino acid degree of conservation in a sequence alignment.

Drawing conclusions on the actual disrupting impact of the detected mutations will only be possible upon enzymatic assays of wild type and mutant proteins or by indirect functional assays such as the confirmation of the response to DM of the genotypes carrying the different variants. Nevertheless, the in silico analysis on the three-dimensional model of DMR6 and DLO proteins can already provide some insights and guide further investigations. Of the six mutations, two (Y89H and I253K) appeared to have a larger impact than the other four on the protein structure and consequently on the enzymatic activity. These changes occurred in amino acids positioned in the hydrophobic core of the protein. They imply the switch from a hydrophobic character to a hydrophilic character of the side chains, which carry a positive charge in the mutated amino acids. The use of a three-dimensional model to map the impacting mutations helped in inferring with a good approximation the position of the amino acids within the structure, in particular whether they are on the protein surface or buried inside the core of the proteins, and whether they are part of beta-structures or alfa-helices. An additional hint of the importance of the Y89 and I253 residues came from the analysis of DMR6 and DLO sequence alignments both within the *Vitis* species, results from this study, as well on a larger set of species. Y89 corresponds to an extremely conserved phenylalanine in other DMR6 and DLO sequences and this is an indication of the importance of an aromatic residue in that position. Interestingly, the amino acid following phenylalanine in several DLO sequences is a histidine. I253 is even more conserved in the sequence alignments and it is only in a few cases substituted by a leucine or a valine, which bear the same chemical properties. This suggests a structural and functional role of this amino acid in that specific position, which would be likely disturbed by the mutation into a lysine, as it was observed in one of the studied genotypes.

### 4.5. Ultimate Application of S Genes

The genetic and protein data observed together with the phenotypic data (Table 2, Figure 2A,B, Figure 3) provide a well-rounded view of the role of the genes scouted here. The *VvDMR6.2* gene arouses a particular interest. The broader genetic analysis allowed us to observe that this gene shows two haplotypes (number 10 and 8) which are more frequently represented in DM resistant genotypes. Through the more focused analysis on the impact of Sanger-confirmed mutations, both haplotypes were found to share the genetic mutation responsible for the amino acid variant E53G. This finding suggests a decisive role of *VvDMR6.2* as *S* gene to grapevine DM and confirms the reliability of the bottleneck analysis here carried out (Figure 1).

Induction of plant defense signaling involves the recognition of specific pathogen effectors by the products of specialized host *R* genes. Numerous plant *R* genes have already been identified and characterized and they are being efficiently used in crop improvement research programs [1]. However, especially in tree species, selection of desirable resistant mutants comes with a cost of lengthy and laborious breeding programs. The effort required to produce resistant plants is often baffled within a few years from the selection because the pathogen evolves mechanisms to circumvent the *R* gene mediated immunity [124,125]. Exploitation of inactive alleles of susceptibility genes seems to be a promising path to introduce effective and durable disease resistance. Since *S* genes’ first discovery [6], converting susceptibility genes in resistance factors has become an increasingly complementary strategy to that of breeding for *R* loci [4], and the advent of new reliable genome editing tools has enhanced this trend. The use of genome editing technologies such as CRISPR-Cas9 allow to specifically and rapidly target susceptibility genes to indirectly obtain resistance in a chosen genetic background, which is highly desired in crops like grapevine where the genetic identity is economically important. Recently, the *S* gene *MdDIPM4* was targeted in apple for a genome editing-driven knock out, resulting in edited plants showing reduced susceptibility to the bacterial pathogen *Erwinia amylovora* [126]. A similar approach was carried out by Low et al. (2020) [127] on *Hv2OGO* gene in barley conferring resistance to *Fusarium graminearum*. However, generation of edited plants and testing of their phenotype still requires years [128,129]. *S* genes may play different functions in the plant, thus pleiotropic effects associated with their knockout may entail a certain fitness cost for the plant. Recently, quantitative regulation of gene expression has been achieved with genome editing on *cis*-regulatory elements [125,130,131] and this might be a strategy to limit negative drawbacks associated with a reduced *S* gene function.

## 5. Conclusions

In this framework, the broad investigation of genetic diversity (until the haplotype level) related to a disease resistance trait presented here has the potential to become a resource in different contexts of plant science, both through the future integration of transcriptomics, proteomics and metabolomics data and as such. The identification of specific homozygous variants in the natural pool can in fact guide genome editing projects in targeting mutations that occur ‘naturally’. This “tailored gene editing” that mimics natural polymorphisms has recently been demonstrated by Bastet et al. (2017, 2019) [132,133]. Finally, breeding programs could benefit from information on selected homozygous and heterozygous *S* gene mutations by implementing a next-generation marker-assisted strategy.

## Figures and Tables

**Table 1:** Investigated genes with Illumina amplicon primers and position.

Gene	ID	Amplicon	Illumina Forward Primer 5′-3′	Illumina Reverse Primer 3′-5′	Amplicon Position
*VvDMR6.1*	VIT_216s0098g00860	1	CTGCTTAGTAGAGTGGTTAT	CGATGTGTTGGATGAGTTGG	Intron-Exon 1 Junction
		2	ATGTCCCCATAATCGACCTC	GTAGAACTCATCGGCCACCT	Exon 1- Intron Junction
		3	ATGGGGTAGCTGCAGAAATG	TTGAAGGAAGGAGGATTGGA	Exon 2
		4	TCTCGAACAAATCCTAATTCAAAA	GAAGAATGGTAAGGGCGTTG	Intron-Exon 3 Junction
		5	AACCCGAGCTCACTTATGGA	AAATTTTAAAAACCGGGCAAA	Exon 3-Intron Junction
		6	GGAAATGGGCATGTGCTAATA	TGCCCCAGAACTTCTTGTAA	Intron-Exon 4 Junction
*VvDMR6.2*	VIT_213s0047g00210	1	TCGGAGTCTTCACTCCCTTT	GCCATAACGGCTACAAGCAT	Exon 1
		2	GGTGTGGATGTGACCAGTGA	CCAAAGGATGGCAATGAAGT	Intron-Exon 2 Junction
		3	AGGAGAAAGTGCACAATTGGA	TCCGAAAAGGAAAAATGATGC	Exon 2-Intron Junction
		4	TCCAAAATGAAGACATAAGAAGGA	TATGTGCTGGCAGTCCGTAA	Intron-Exon 3 Junction
		5	CTTGTCCCGAGCCAGAGTTA	CCTGCATGCAATCATTTGTT	Exon 3-Intron Junction
		6	CCCAGGTGCTTTTGTTGTTA	CCCTTGCTGGACTAATGAGC	Exon 3- Exon 4 Junction
		7	CGATTGCTTCTTTCCTCTGC	CGCATTATGCCTTGTTGAAG	Exon 4
*VvDLO1*	VIT_215s0048g02430	1	ACAGGCCATCCCTCAGTACA	ATCGACATGTACCCGAAAAA	Exon 1
		2	CCTTGCTTTGACATGATTCTTC	TGAAAGATGGAGGGTTGGAG	Exon 2
		3	CCAACTGGAGAGATTTCCTGA	CGCCTTATCTATGTGGTTCCTC	Exon 2- Exon 3 Junction
		4	CTGGCCATGCTGATCCTAAT	CCTATGGACCGCACTCTTGT	Exon 3- Exon 4 Junction
		5	TTCCTGTAAAGGGCAGGATG	TTCCTGTAAAGGGCAGGATG	Exon 3- Exon 4 Junction
*VvDLO2*	VIT_202s0025g02970	1	CAACCCCCACTTGTGAATTT	CTTGGCCAATCTGTTTGACA	Intron-Exon 1 Junction
		2	AAGGATGTCCAGGCATCAGA	GAGCCTGACTGGATTGGAAG	Exon 1
		3	AGCTGCCAGAAAGCGAGA	CATGTAACTGCATGTTGGTCAG	Exon 1-Intron Junction
		4	TCTGACCAACATGCAGTTACA	TCTTGGAGAAGAACTGTGATTAAA	Intron-Exon 2 Junction
		5	CTTATGGGTTGCCTGGACAT	TTTTCCTCATTTTTGCAGGTG	Exon 2-Intron Junction

**Table 2:** Example of the haplotypic structure for each analyzed genotype.

Genotype	Taxon	*VvDMR6.1* Haplotype	*VvDMR6.2* Haplotype	*VvDLO1* Haplotype	*VvDLO2* Haplotype	OIV 452(-1)
PN40024		1,1	1,1	1,1	1,1	
B87-60	*Vitis* hybrid	5,8	-	-	-	
Blanc du Bois	*Vitis* hybrid	15,16	-	-	-	6 †
Blue Lake	*Vitis* hybrid	5,8	8,13	-	-	8 †
Captivator	*Vitis* hybrid	-	7,8	-	-	7 †
Catawba	*Vitis* hybrid	-	1,3	-	-	3 ‡
Chancellor	*Vitis* hybrid	1,17	-	-	1,19	1 ‡
Clinton	*Vitis* hybrid	-	10,10	-	7,14	1 †
D’Arpa	*Vitis* hybrid	-	7,8	-	-	9 †
Diamond	*Vitis* hybrid	-	-	-	1,19	5 ‡
F560 Big Brown	*Vitis* hybrid	-	-	8,9	-	9 †
FLA 449	*Vitis* hybrid	-	1,10	-	-	
FLA W1521	*Vitis* hybrid	-	8,8	-	-	8 †
Golden Muscat	*Vitis* hybrid	-	5,10	-	1,14	2 ‡
Herbert	*Vitis* hybrid	-	5,10	1,3	-	
Kunleany	*Vitis* hybrid	-	-	1,7	-	9 †
Lenoir	*Vitis* hybrid	-	-	9,9	-	8 †
M11-14St. George	*Vitis* hybrid	-	-	-	1,6	9 †
Mantey	*Vitis* hybrid	-	-	2,2	-	
Mars	*Vitis* hybrid	-	1,9	-	-	8 †
MW66	*Vitis* hybrid	2,3	-	-	-	5 †
NY08.0701b	*Vitis* hybrid	12,14	-	-	-	
NY63.1016.01	*Vitis* hybrid	11,13	-	-	-	
NY65.0562.01	*Vitis* hybrid	-	-	-	1,15	
NY84.0100.05	*Vitis* hybrid	1,13	-	-	-	
NY97.0503.02	*Vitis* hybrid	-	-	-	7,14	
NY97.0512.01	*Vitis* hybrid	-	1,4	-	1,17	
Ontario	*Vitis* hybrid	-	-	-	1,4	5 ‡
Petra	*Vitis* hybrid	7,9	1,6	4,5	-	9 †
Pixiola	*Vitis* hybrid	-	-	-	1,18	
Schuyler	*Vitis* hybrid	-	-	-	1,14	5†
Seibel 880	*Vitis* hybrid	-	-	-	1,14	
Sheridan	*Vitis* hybrid	-	10,10	-	-	
Steuben	*Vitis* hybrid	-	-	-	1,5	2 ‡
*V. riparia x V. cordifolia*	*Vitis* hybrid	-	-	-	11,14	
Venus	*Vitis* hybrid	-	5,8	-	-	7 †
Wayne	*Vitis* hybrid	-	5,10	-	-	
Worden	*Vitis* hybrid	-	10,10	-	1,16	
*V. aestivalis*	*Vitis spp.*	-	-	-	10,18	9 ‡
*V. berlandieri* Texas	*Vitis spp.*	-	-	4,4	8,9	9 †
*V. cordifolia*	*Vitis spp.*	-	-	1,4	8,19	9 †
*V. rubra*	*Vitis spp.*	-	1,12	-	-	9 †
*V. rupestris* du Lot	*Vitis spp.*	4,10	-	-	-	9 †
*V. smalliana*	*Vitis spp.*	-	-	6,1	1,19	
Coia1	*Vitis spp.*/hybrid	-	10,11	-	-	9 †
Coia5	*Vitis spp.*/hybrid	-	10,10	-	-	9 †
Coia7	*Vitis spp.*/hybrid	-	10,14	-	7,19	9 †
Coia9	*Vitis spp.*/hybrid	-	10,1	-	-	9 †
Coia10	*Vitis spp.*/hybrid	-	10,10	-	-	9 †
Coia11	*Vitis spp.*/hybrid	-	10,10	-	-	9 †
Coia12	*Vitis spp.*/hybrid	-	10,10	-	3,19	not available
Corella2	*Vitis spp.*/hybrid	-	-	-	1,18	not available
Lorenzo1	*Vitis spp.*/hybrid	-	-	-	12,13	9 †
Franconia	*Vitis vinifera*	-	-	-	1,2	1 †
Italia	*Vitis vinifera*	-	1,2	-	-	1 †
Pinot gris	*Vitis vinifera*	6,15	-	-	-	1 †

OIV, International Organisation of Vine and Wine; †: unpublished data; ‡: OIV-452(-1) scores provided by Cadle-Davidson (2008) [85].

## Data Availability

The data presented in this study are available in Supplementary Materials.

## Supplementary Materials

The following are available online at https://www.mdpi.com/2218-273X/11/2/181/s1: Figure S1. CLUSTALW alignment of bonafide and putative DMR6 and DLO proteins from different species, Table S1. List of studied grapevine genotypes, Table S2. Selected genotypes for Sanger sequencing of each gene, investigated variants with their physical position, and sequencing primers, Table S3. List of impacting mutations with positions and data in VCF (Variant Call Format), Table S4. List of genotypes showing impacting mutations—heterozygous (He) or homozygous (Ho) status—in at least one gene, Table S5. Haplotype identification and frequencies determined for the VvDMR6.1, VvDMR6.2, VvDLO1, VvDLO2 genes.

## References

[1] Gururani M.A., Venkatesh J., Upadhyaya C.P., Nookaraju A., Pandey S.K., Park S.W. (2012). Plant disease resistance genes: Current status and future directions. Physiol. Mol. Plant Pathol..

[2] Jones J.D.G., Dangl J.L. (2006). The plant immune system. Nature.

[3] Meyers B.C., Kaushik S., Nandety R.S. (2005). Evolving disease resistance genes. Curr. Opin. Plant Biol..

[4] Van Schie C.C.N., Takken F.L.W. (2014). Susceptibility genes 101: How to be a good host. Annu. Rev. Phytopathol..

[5] Fawke S., Doumane M., Schornack S. (2015). Oomycete Interactions with Plants: Infection Strategies and Resistance Principles. Microbiol. Mol. Biol. Rev..

[6] Jorgensen J.H. (1992). Discovery, characterization and exploitation of Mlo powdery mildew. Euphytica.

[7] Kusch S., Panstruga R. (2017). mlo-Based Resistance: An Apparently Universal “Weapon” to Defeat Powdery Mildew Disease. Mol. Plant Microbe Interact..

[8] Van Damme M., Andel A., Huibers R.P., Panstruga R., Weisbeek P.J., Van Den Ackerveken G. (2005). Identification of Arabidopsis loci required for susceptibility to the downy mildew pathogen Hyaloperonospora parasitica. Mol. Plant Microbe Interact..

[9] Van Damme M., Huibers R.P., Elberse J., Van Den Ackerveken G. (2008). Arabidopsis DMR6 encodes a putative 2OG-Fe(II) oxygenase that is defense-associated but required for susceptibility to downy mildew. Plant J..

[10] Zhang K., Halitschke R., Yin C., Liu C.-J., Gan S.-S. (2013). Salicylic acid 3-hydroxylase regulates *Arabidopsis* leaf longevity by mediating salicylic acid catabolism. Proc. Natl. Acad. Sci. USA.

[11] Zeilmaker T., Ludwig N.R., Elberse J., Seidl M.F., Berke L., Van Doorn A., Schuurink R.C., Snel B., Van Den Ackerveken G. (2015). Downy mildew resistant 6 and DMR6-like oxygenase 1 are partially redundant but distinct suppressors of immunity in Arabidopsis. Plant J..

[12] Zhang Y.J., Zhao L., Zhao J.Z., Li Y.J., Wang J.B., Guo R., Gan S.S., Liu C.J., Zhanga K.W. (2017). S5H/DMR6 encodes a salicylic acid 5-hydroxylase that fine-tunes salicylic acid homeostasis. Plant Physiol..

[13] De Toledo Thomazella D.P., Brail Q., Dahlbeck D., Staskawicz B.J. (2016). CRISPR-Cas9 mediated mutagenesis of a DMR6 ortholog in tomato confers broad-spectrum disease resistance. bioRxiv.

[14] Schouten H.J., Krauskopf J., Visser R.G.F., Bai Y. (2014). Identification of candidate genes required for susceptibility to powdery or downy mildew in cucumber. Euphytica.

[15] Sun K., van Tuinen A., van Kan J.A.L., Wolters A.M.A., Jacobsen E., Visser R.G.F., Bai Y. (2017). Silencing of DND1 in potato and tomato impedes conidial germination, attachment and hyphal growth of Botrytis cinerea. BMC Plant Biol..

[16] Zhang W., Mirlohi S., Li X., He Y. (2018). Identification of functional single-nucleotide polymorphisms affecting leaf hair number in Brassica rapa. Plant Physiol..

[17] Ganal M.W., Altmann T., Röder M.S. (2009). SNP identification in crop plants. Curr. Opin. Plant Biol..

[18] Brookes A.J. (1999). The essence of SNPs [Review]. Gene.

[19] Andersen J.R., Lübberstedt T. (2003). Functional markers in plants. Trends Plant Sci..

[20] Polanco C., Sáenz de Miera L.E., González A.I., García P., Fratini R., Vaquero F., Javier Vences F., De La Vega M.P. (2019). Construction of a high-density interspecific (*Lens culinaris* × *L. Odemensis*) genetic map based on functional markers for mapping morphological and agronomical traits, and QTLs affecting resistance to Ascochyta in lentil. PLoS ONE.

[21] Burow G., Chopra R., Sattler S., Burke J., Acosta-Martinez V., Xin Z. (2019). Deployment of SNP (CAPS and KASP) markers for allelic discrimination and easy access to functional variants for brown midrib genes bmr6 and bmr12 in Sorghum bicolor. Mol. Breed..

[22] Fan C., Yu S., Wang C., Xing Y. (2009). A causal C-A mutation in the second exon of GS3 highly associated with rice grain length and validated as a functional marker. Theor. Appl. Genet..

[23] Yang Y., Zhang H., Xuan N., Chen G., Liu X., Yao F., Ding H. (2017). Identification of blast resistance genes in 358 rice germplasms (*Oryza sativa* L.) using functional molecular markers. Eur. J. Plant Pathol..

[24] Edwards D., Forster J.W., Cogan N.O., Batley J., Chagné D. (2007). Single nucleotide polymorphism discovery. Association Mapping in Plants.

[25] Varshney R.K., Nayak S.N., May G.D., Jackson S.A. (2009). Next-generation sequencing technologies and their implications for crop genetics and breeding. Trends Biotechnol..

[26] Schadt E.E., Turner S., Kasarskis A. (2010). A window into third-generation sequencing. Hum. Mol. Genet..

[27] Arabidopsis Genome Initiative (2000). Analysis of the genome sequence of the flowering plant Arabidopsis thaliana. Nature.

[28] Ching A., Caldwell K.S., Jung M., Dolan M., Smith O.S.H., Tingey S., Morgante M., Rafalski A.J. (2002). SNP frequency, haplotype structure and linkage disequilibrium in elite maize inbred lines. BMC Genet..

[29] Atwell S., Huang Y.S., Vilhjálmsson B.J., Willems G., Horton M., Li Y., Meng D., Platt A., Tarone A.M., Hu T.T. (2010). Genome-wide association study of 107 phenotypes in Arabidopsis thaliana inbred lines. Nature.

[30] Xu X., Liu X., Ge S., Jensen J.D., Hu F., Li X., Dong Y., Gutenkunst R.N., Fang L., Huang L. (2012). Resequencing 50 accessions of cultivated and wild rice yields markers for identifying agronomically important genes. Nat. Biotechnol..

[31] Raman H., Dalton-Morgan J., Diffey S., Raman R., Alamery S., Edwards D., Batley J. (2014). SNP markers-based map construction and genome-wide linkage analysis in Brassica napus. Plant Biotechnol. J..

[32] Vos P.G., Uitdewilligen J.G.A.M.L., Voorrips R.E., Visser R.G.F., van Eck H.J. (2015). Development and analysis of a 20K SNP array for potato (Solanum tuberosum): An insight into the breeding history. Theor. Appl. Genet..

[33] Hulse-Kemp A.M., Ashrafi H., Plieske J., Lemm J., Stoffel K., Hill T., Luerssen H., Pethiyagoda C.L., Lawley C.T., Ganal M.W. (2016). A HapMap leads to a Capsicum annuum SNP infinium array: A new tool for pepper breeding. Hortic. Res..

[34] Peterson G.W., Dong Y., Horbach C., Fu Y.B. (2014). Genotyping-by-sequencing for plant genetic diversity analysis: A lab guide for SNP genotyping. Diversity.

[35] Campbell N.R., Harmon S.A., Narum S.R. (2015). Genotyping-in-Thousands by sequencing (GT-seq): A cost effective SNP genotyping method based on custom amplicon sequencing. Mol. Ecol. Resour..

[36] Kumar S., Banks T.W., Cloutier S. (2012). SNP discovery through next-generation sequencing and its applications. Int. J. Plant Genomics.

[37] Durstewitz G., Polley A., Plieske J., Luerssen H., Graner E.M., Wieseke R., Ganal M.W. (2010). SNP discovery by amplicon sequencing and multiplex SNP genotyping in the allopolyploid species Brassica napus. Genome.

[38] Yang S., Fresnedo-Ramírez J., Wang M., Cote L., Schweitzer P., Barba P., Takacs E.M., Clark M., Luby J., Manns D.C. (2016). A next-generation marker genotyping platform (AmpSeq) in heterozygous crops: A case study for marker-assisted selection in grapevine. Hortic. Res..

[39] Cho Y.B., Jones S.I., Vodkin L.O. (2017). Mutations in Argonaute5 illuminate epistatic interactions of the K1 and I loci leading to saddle seed color patterns in glycine max. Plant Cell.

[40] Shimray P.W., Bajaj D., Srivastava R., Daware A., Upadhyaya H.D., Kumar R., Bharadwaj C., Tyagi A.K., Parida S.K. (2017). Identifying Transcription Factor Genes Associated with Yield Traits in Chickpea. Plant Mol. Biol. Report..

[41] Hong Y., Liao D., Hu A., Wang H., Chen J., Khan S., Su J., Li H. (2015). Diversity of endophytic and rhizoplane bacterial communities associated with exotic Spartina alterniflora and native mangrove using Illumina amplicon sequencing. Can. J. Microbiol..

[42] Kinoti W.M., Constable F.E., Nancarrow N., Plummer K.M., Rodoni B. (2017). Analysis of intra-host genetic diversity of Prunus necrotic ringspot virus (PNRSV) using amplicon next generation sequencing. PLoS ONE.

[43] Gupta P.K., Roy J.K., Prasad M. (2001). Single nucleotide polymorphisms: A new paradigm for molecular marker technology and DNA polymorphism detection with emphasis on their use in plants. Curr. Sci..

[44] Bianco L., Cestaro A., Linsmith G., Muranty H., Denancé C., Théron A., Poncet C., Micheletti D., Kerschbamer E., Di Pierro E.A. (2016). Development and validation of the Axiom®Apple480K SNP genotyping array. Plant J..

[45] Marrano A., Martínez-García P.J., Bianco L., Sideli G.M., Di Pierro E.A., Leslie C.A., Stevens K.A., Crepeau M.W., Troggio M., Langley C.H. (2019). A new genomic tool for walnut (*Juglans regia* L.): Development and validation of the high-density Axiom^TM^ J. regia 700K SNP genotyping array. Plant Biotechnol. J..

[46] Hardner C.M., Hayes B.J., Kumar S., Vanderzande S., Cai L., Piaskowski J., Quero-Garcia J., Campoy J.A., Barreneche T., Giovannini D. (2019). Prediction of genetic value for sweet cherry fruit maturity among environments using a 6K SNP array. Hortic. Res..

[47] Li X., Singh J., Qin M., Li S., Zhang X., Zhang M., Khan A., Zhang S., Wu J. (2019). Development of an integrated 200K SNP genotyping array and application for genetic mapping, genome assembly improvement and genome wide association studies in pear (Pyrus). Plant Biotechnol. J..

[48] Merot-L’anthoene V., Tournebize R., Darracq O., Rattina V., Lepelley M., Bellanger L., Tranchant-Dubreuil C., Coulée M., Pégard M., Metairon S. (2019). Development and evaluation of a genome-wide Coffee 8.5K SNP array and its application for high-density genetic mapping and for investigating the origin of *Coffea arabica* L.. Plant Biotechnol. J..

[49] Mercati F., De Lorenzis G., Brancadoro L., Lupini A., Abenavoli M.R., Barbagallo M.G., Di Lorenzo R., Scienza A., Sunseri F. (2016). High-throughput 18K SNP array to assess genetic variability of the main grapevine cultivars from Sicily. Tree Genet. Genomes.

[50] Laucou V., Launay A., Bacilieri R., Lacombe T., Adam-Blondon A.F., Bérard A., Chauveau A., De Andrés M.T., Hausmann L., Ibáñez J. (2018). Extended diversity analysis of cultivated grapevine *Vitis vinifera* with 10K genome-wide SNPs. PLoS ONE.

[51] Feuillet C., Leach J.E., Rogers J., Schnable P.S., Eversole K. (2011). Crop genome sequencing: Lessons and rationales. Trends Plant Sci..

[52] Bolger M.E., Weisshaar B., Scholz U., Stein N., Usadel B., Mayer K.F.X. (2014). Plant genome sequencing—Applications for crop improvement. Curr. Opin. Biotechnol..

[53] Owens C.L. (2003). SNP detection and genotyping in Vitis. Acta Hortic..

[54] Jaillon O. (2007). The grapevine genome sequence suggests ancestral hexaploidization in major angiosperm phyla. Nature.

[55] Velasco R., Zharkikh A., Troggio M., Cartwright D.A., Cestaro A., Pruss D., Pindo M., FitzGerald L.M., Vezzulli S., Reid J. (2007). A High Quality Draft Consensus Sequence of the Genome of a Heterozygous Grapevine Variety. PLoS ONE.

[56] Carrier G., Le Cunff L., Dereeper A., Legrand D., Sabot F., Bouchez O., Audeguin L., Boursiquot J.M., This P. (2012). Transposable elements are a major cause of somatic polymorphism in *Vitis vinifera* L.. PLoS ONE.

[57] Gambino G., Dal Molin A., Boccacci P., Minio A., Chitarra W., Avanzato C.G., Tononi P., Perrone I., Raimondi S., Schneider A. (2017). Whole-genome sequencing and SNV genotyping of “Nebbiolo” (*Vitis vinifera* L.) clones. Sci. Rep..

[58] Roach M.J., Johnson D.L., Bohlmann J., van Vuuren H.J.J., Jones S.J.M., Pretorius I.S., Schmidt S.A., Borneman A.R. (2018). Population sequencing reveals clonal diversity and ancestral inbreeding in the grapevine cultivar Chardonnay. PLoS Genet..

[59] Minio A., Massonnet M., Figueroa-Balderas R., Castro A., Cantu D. (2019). Diploid genome assembly of the wine grape carménère. Genes Genomes Genet..

[60] Girollet N., Rubio B., Bert P.-F. (2019). De novo phased assembly of the Vitis riparia grape genome. Sci. Data.

[61] Cochetel N., Minio A., Vondras A.M., Figueroa-Balderas R., Cantu D. (2020). Diploid chromosome-scale assembly of the Muscadinia rotundifolia genome supports chromosome fusion and disease resistance gene expansion during Vitis and Muscadinia divergence. bioRxiv.

[62] Topfer R., Hausmann L. (2010). Table of Loci for Traits in Grapevine Relevant for Breeding and Genetics. VIVC Vitis Int. Var. Cat..

[63] Sargolzaei M., Maddalena G., Bitsadze N., Maghradze D., Bianco P.A., Failla O., Toffolatti S.L., Lorenzis G. (2020). De Rpv29, Rpv30 and Rpv31: Three Novel Genomic Loci Associated With Resistance to Plasmopara viticola in *Vitis vinifera*. Front. Plant Sci..

[64] Barba P., Cadle-Davidson L., Harriman J., Glaubitz J.C., Brooks S., Hyma K., Reisch B. (2014). Grapevine powdery mildew resistance and susceptibility loci identified on a high-resolution SNP map. Theor. Appl. Genet..

[65] Winterhagen P., Howard S.F., Qiu W., Kovács L.G. (2008). Transcriptional up-regulation of grapevine MLO genes in response to powdery mildew infection. Am. J. Enol. Vitic..

[66] Feechan A., Jermakow A.M., Dry I.B. (2009). Grapevine MLO candidates required for powdery mildew pathogenicity?. Plant Signal. Behav..

[67] Pessina S., Lenzi L., Perazzolli M., Campa M., Dalla Costa L., Urso S., Valè G., Salamini F., Velasco R., Malnoy M. (2016). Knockdown of MLO genes reduces susceptibility to powdery mildew in grapevine. Hortic. Res..

[68] Madeira F., Park Y.M., Lee J., Buso N., Gur T., Madhusoodanan N., Basutkar P., Tivey A.R.N., Potter S.C., Finn R.D. (2019). The EMBL-EBI search and sequence analysis tools APIs in 2019. Nucleic Acids Res..

[69] Vitulo N., Forcato C., Carpinelli E., Telatin A., Campagna D., D’Angelo M., Zimbello R., Corso M., Vannozzi A., Bonghi C. (2014). A deep survey of alternative splicing in grape reveals changes in the splicing machinery related to tissue, stress condition and genotype. BMC Plant Biol..

[70] Canaguier A., Grimplet J., Di Gaspero G., Scalabrin S., Duchêne E., Choisne N., Mohellibi N., Guichard C., Rombauts S., Le Clainche I. (2017). A new version of the grapevine reference genome assembly (12X.v2) and of its annotation (VCost.v3). Genomics Data.

[71] Li H., Durbin R. (2010). Fast and accurate long-read alignment with Burrows–Wheeler transform. Bioinformatics.

[72] Staden R., Beal K.F., Bonfield J.K. (2000). The staden package, 1998. Bioinformatics Methods and Protocols.

[73] Li H., Handsaker B., Wysoker A., Fennell T., Ruan J., Homer N., Marth G., Abecasis G., Durbin R. (2009). The Sequence Alignment/Map format and SAMtools. Bioinformatics.

[74] Danecek P., Auton A., Abecasis G., Albers C.A., Banks E., DePristo M.A., Handsaker R.E., Lunter G., Marth G.T., Sherry S.T. (2011). The variant call format and VCFtools. Bioinformatics.

[75] Cingolani P., Platts A., Wang L.L., Coon M., Nguyen T., Wang L., Land S.J., Lu X., Ruden D.M. (2012). A program for annotating and predicting the effects of single nucleotide polymorphisms, SnpEff. Fly.

[76] Kumar P., Henikoff S., Ng P.C. (2009). Predicting the effects of coding non-synonymous variants on protein function using the SIFT algorithm. Nat. Protoc..

[77] Betts M.J., Russel R.B. (2003). Amino acid properties and consequences of substitutions. Bioinformatics for Geneticists.

[78] Stephens M., Smith N.J., Donnelly P. (2001). A new statistical method for haplotype reconstruction from population data. Am. J. Hum. Genet..

[79] OIV (International Organisation of Vine and Wine) (2009). OIV Descriptor List for Grape Varieties and Vitis Species.

[80] Zeilmaker T. (2012). Functional and Applied Aspects of the DOWNY MILDEW RESISTANT 1 and 6 Genes in Arabidopsis. Ph.D. Thesis.

[81] Proost S., Van Bel M., Vaneechoutte D., Van De Peer Y., Inzé D., Mueller-Roeber B., Vandepoele K. (2015). PLAZA 3.0: An access point for plant comparative genomics. Nucleic Acids Res..

[82] Zimmermann L., Stephens A., Nam S.Z., Rau D., Kübler J., Lozajic M., Gabler F., Söding J., Lupas A.N., Alva V. (2018). A Completely Reimplemented MPI Bioinformatics Toolkit with a New HHpred Server at its Core. J. Mol. Biol..

[83] Kluza A., Niedzialkowska E., Kurpiewska K., Wojdyla Z., Quesne M., Kot E., Porebski P.J., Borowski T. (2018). Crystal structure of thebaine 6-O-demethylase from the morphine biosynthesis pathway. J. Struct. Biol..

[84] Amrine K.C.H., Blanco-Ulate B., Riaz S., Pap D., Jones L., Figueroa-Balderas R., Walker M.A., Cantu D. (2015). Comparative transcriptomics of Central Asian *Vitis vinifera* accessions reveals distinct defense strategies against powdery mildew. Hortic. Res..

[85] Cadle-Davidson L. (2008). Variation within and between Vitis spp. for foliar resistance to the downy mildew pathogen Plasmopara viticola. Plant Dis..

[86] Lijavetzky D., Cabezas J., Ibáñez A., Rodríguez V., Martínez-Zapater J.M. (2007). High throughput SNP discovery and genotyping in grapevine (*Vitis vinifera* L.) by combining a re-sequencing approach and SNPlex technology. BMC Genomics.

[87] Vezzulli S., Micheletti D., Riaz S., Pindo M., Viola R., This P., Walker M.A., Troggio M., Velasco R. (2008). A SNP transferability survey within the genus Vitis. BMC Plant Biol..

[88] Vezzulli S., Troggio M., Coppola G., Jermakow A., Cartwright D., Zharkikh A., Stefanini M., Grando M.S., Viola R., Adam-Blondon A.F. (2008). A reference integrated map for cultivated grapevine (*Vitis vinifera* L.) from three crosses, based on 283 SSR and 501 SNP-based markers. Theor. Appl. Genet..

[89] Salmaso M., Faes G., Segala C., Stefanini M., Salakhutdinov I., Zyprian E., Toepfer R., Grando M.S., Velasco R. (2004). Genome diversity and gene haplotypes in the grapevine (Vitis. Mol. Breed..

[90] Marrano A., Birolo G., Prazzoli M.L., Lorenzi S., Valle G., Grando M.S. (2017). SNP-discovery by RAD-sequencing in a germplasm collection of wild and cultivated grapevines (*V. vinifera* L.). PLoS ONE.

[91] Schneider K., Weisshaar B., Borchardt D.C., Salamini F. (2001). SNP frequency and allelic haplotype structure of Beta vulgaris expressed genes. Mol. Breed..

[92] Simko I., Haynes K.G., Jones R.W. (2006). Assessment of linkage disequilibrium in potato genome with single nucleotide polymorphism markers. Genetics.

[93] Byers R.L., Harker D.B., Yourstone S.M., Maughan P.J., Udall J.A. (2012). Development and mapping of SNP assays in allotetraploid cotton. Theor. Appl. Genet..

[94] Zhu Y.L., Song Q.J., Hyten D.L., Van Tassell C.P., Matukumalli L.K., Grimm D.R., Hyatt S.M., Fickus E.W., Young N.D., Cregan P.B. (2003). Single-nucleotide polymorphisms in soybean. Genetics.

[95] Wu S.B., Wirthensohn M.G., Hunt P., Gibson J.P., Sedgley M. (2008). High resolution melting analysis of almond SNPs derived from ESTs. Theor. Appl. Genet..

[96] Salmaso M., Malacarne G., Troggio M., Faes G., Stefanini M., Grando M.S., Velasco R. (2008). A grapevine (*Vitis vinifera* L.) genetic map integrating the position of 139 expressed genes. Theor. Appl. Genet..

[97] Aranzana M.J., Illa E., Howad W., Arús P. (2012). A first insight into peach [Prunus persica (L.) Batsch] SNP variability. Tree Genet. Genomes.

[98] Tuskan G.A., Difazio S., Jansson S., Bohlmann J., Grigoriev I., Hellsten U., Putnam N., Ralph S., Rombauts S., Salamov A. (2006). The Genome of Black Cottonwood Populus trichocarpa (Torr. & Gray). Science.

[99] Thavamanikumar S., McManus L.J., Tibbits J.F.G., Bossinger G. (2011). The significance of single nucleotide polymorphisms (SNPs) in Eucalyptus globulus breeding programs. Aust. For..

[100] Emanuelli F., Lorenzi S., Grzeskowiak L., Catalano V., Stefanini M., Troggio M., Myles S., Martinez-Zapater J.M., Zyprian E., Moreira F.M. (2013). Genetic diversity and population structure assessed by SSR and SNP markers in a large germplasm collection of grape. BMC Plant Biol..

[101] Jones E.S., Sullivan H., Bhattramakki D., Smith J.S.C. (2007). A comparison of simple sequence repeat and single nucleotide polymorphism marker technologies for the genotypic analysis of maize (*Zea mays* L.). Theor. Appl. Genet..

[102] Grattapaglia D., Silva-Junior O.B., Kirst M., de Lima B.M., Faria D.A., Pappas G.J. (2011). High-throughput SNP genotyping in the highly heterozygous genome of Eucalyptus: Assay success, polymorphism and transferability across species. BMC Plant Biol..

[103] Biswas C., Dey P., Karmakar P.G., Satpathy S. (2015). Discovery of large-scale SNP markers and construction of linkage map in a RIL population of jute (*Corchorus capsularis*). Mol. Breed..

[104] Cheng L., Chen X., Jiang C., Ma B., Ren M., Cheng Y., Liu D., Geng R., Yang A. (2019). High-density SNP genetic linkage map construction and quantitative trait locus mapping for resistance to cucumber mosaic virus in tobacco (*Nicotiana tabacum* L.). Crop J..

[105] Aflitos S., Schijlen E., De Jong H., De Ridder D., Smit S., Finkers R., Wang J., Zhang G., Li N., Mao L. (2014). Exploring genetic variation in the tomato (Solanum section Lycopersicon) clade by whole-genome sequencing. Plant J..

[106] Xanthopoulou A., Montero-Pau J., Mellidou I., Kissoudis C., Blanca J., Picó B., Tsaballa A., Tsaliki E., Dalakouras A., Paris H.S. (2019). Whole-genome resequencing of Cucurbita pepo morphotypes to discover genomic variants associated with morphology and horticulturally valuable traits. Hortic. Res..

[107] Dong X., Wang Z., Tian L., Zhang Y., Qi D., Huo H., Xu J., Li Z., Liao R., Shi M. (2019). De novo assembly of a wild pear (*Pyrus betuleafolia*) genome. Plant Biotechnol. J..

[108] Cardone M.F., D’Addabbo P., Alkan C., Bergamini C., Catacchio C.R., Anaclerio F., Chiatante G., Marra A., Giannuzzi G., Perniola R. (2016). Inter-varietal structural variation in grapevine genomes. Plant J..

[109] Baudhuin L.M., Lagerstedt S.A., Klee E.W., Fadra N., Oglesbee D., Ferber M.J. (2015). Confirming variants in next-generation sequencing panel testing by sanger sequencing. J. Mol. Diagnostics.

[110] Mu W., Lu H.M., Chen J., Li S., Elliott A.M. (2016). Sanger Confirmation Is Required to Achieve Optimal Sensitivity and Specificity in Next-Generation Sequencing Panel Testing. J. Mol. Diagnostics.

[111] Quaynor S.D., Bosley M.E., Duckworth C.G., Porter K.R., Kim S.H., Kim H.G., Chorich L.P., Sullivan M.E., Choi J.H., Cameron R.S. (2016). Targeted next generation sequencing approach identifies eighteen new candidate genes in normosmic hypogonadotropic hypogonadism and Kallmann syndrome. Mol. Cell. Endocrinol..

[112] Strom S.P., Lee H., Das K., Vilain E., Nelson S.F., Grody W.W., Deignan J.L. (2014). Assessing the necessity of confirmatory testing for exome-sequencing results in a clinical molecular diagnostic laboratory. Genet. Med..

[113] Zheng J., Zhang H., Banerjee S., Li Y., Zhou J., Yang Q., Tan X., Han P., Fu Q., Cui X. (2019). A comprehensive assessment of Next-Generation Sequencing variants validation using a secondary technology. Mol. Genet. Genomic Med..

[114] Satya R.V., DiCarlo J. (2014). Edge effects in calling variants from targeted amplicon sequencing. BMC Genomics.

[115] Lacombe T., Audeguin L., Boselli M., Bucchetti B., Cabello F., Chatelet P., Crespan M., D’Onofrio C., Eiras Dias J., Ercisli S. (2011). Grapevine European Catalogue: Towards a comprehensive list. Vitis.

[116] Excoffier L., Langaney A. (1989). Origin and Differentiation of Human Mitochondrial DNA. J. Hum. Genet..

[117] Watterson G.A., Guess H.A. (1977). Is the most frequent allele the oldest?. Theor. Popul. Biol..

[118] Donnelly P., Tavaré S. (1986). The ages of alleles and a coalescent. Adv. Appl. Probab..

[119] Riahi L., Zoghlami N., Dereeper A., Laucou V., Mliki A., This P. (2013). Single nucleotide polymorphism and haplotype diversity of the gene NAC4 in grapevine. Ind. Crops Prod..

[120] Fernandez L., Le Cunff L., Tello J., Lacombe T., Boursiquot J.M., Fournier-Level A., Bravo G., Lalet S., Torregrosa L., This P. (2014). Haplotype diversity of VvTFL1A gene and association with cluster traits in grapevine (*V. vinifera*). BMC Plant Biol..

[121] Nicolas S.D., Péros J.P., Lacombe T., Launay A., Le Paslier M.C., Bérard A., Mangin B., Valière S., Martins F., Le Cunff L. (2016). Genetic diversity, linkage disequilibrium and power of a large grapevine (*Vitis vinifera* L) diversity panel newly designed for association studies. BMC Plant Biol..

[122] Magris G., Di Gaspero G., Marroni F., Zenoni S., Tornielli G.B., Celii M., De Paoli E., Pezzotti M., Conte F., Paci P. (2019). Genetic, epigenetic and genomic effects on variation of gene expression among grape varieties. Plant J..

[123] Foria S., Copetti D., Eisenmann B., Magris G., Vidotto M., Scalabrin S., Testolin R., Cipriani G., Wiedemann-Merdinoglu S., Bogs J. (2020). Gene duplication and transposition of mobile elements drive evolution of the Rpv3 resistance locus in grapevine. Plant J..

[124] Schaart J.G., van de Wiel C.C.M., Lotz L.A.P., Smulders M.J.M. (2016). Opportunities for Products of New Plant Breeding Techniques. Trends Plant Sci..

[125] Bisht D.S., Bhatia V., Bhattacharya R. (2019). Improving plant-resistance to insect-pests and pathogens: The new opportunities through targeted genome editing. Semin. Cell Dev. Biol..

[126] Pompili V., Dalla Costa L., Piazza S., Pindo M., Malnoy M. (2020). Reduced fire blight susceptibility in apple cultivars using a high-efficiency CRISPR/Cas9-FLP/FRT-based gene editing system. Plant Biotechnol. J..

[127] Low Y.C., Lawton M.A., Di R. (2020). Validation of barley 2OGO gene as a functional orthologue of Arabidopsis DMR6 gene in Fusarium head blight susceptibility. Sci. Rep..

[128] Ffrench-Constant R.H., Bass C. (2017). Does resistance really carry a fitness cost?. Curr. Opin. Insect Sci..

[129] Zaidi S.S.E.A., Mukhtar M.S., Mansoor S. (2018). Genome Editing: Targeting Susceptibility Genes for Plant Disease Resistance. Trends Biotechnol..

[130] Rodríguez-Leal D., Lemmon Z.H., Man J., Bartlett M.E., Lippman Z.B. (2017). Engineering Quantitative Trait Variation for Crop Improvement by Genome Editing. Cell.

[131] Wolter F., Puchta H. (2018). Application of CRISPR/Cas to Understand Cis- and Trans-Regulatory Elements in Plants. Methods in Molecular Biology.

[132] Bastet A., Robaglia C., Gallois J.L. (2017). eIF4E Resistance: Natural Variation Should Guide Gene Editing. Trends Plant Sci..

[133] Bastet A., Zafirov D., Giovinazzo N., Guyon-Debast A., Nogué F., Robaglia C., Gallois J.-L. (2019). Mimicking natural polymorphism in eIF4E by CRISPR-Cas9 base editing is associated with resistance to potyviruses. Plant Biotechnol. J..

