# Supplementary material for: Mining Grapevine Downy Mildew Susceptibility Genes: A Resource for Genomics-Based Breeding and Tailored Gene Editing
Source: Biomolecules. 2021 Jan 28;11(2):181. doi: 10.3390/biom11020181 (PMC7912118; doi:10.3390/biom11020181)
Supplement: Supplementary file 1 [file biomolecules-11-00181-s001.zip › Supplementary_files_DEF/Figure_S1_25.01.21.pdf]

```
DMR6
B.distachyon_Bradi5g19240        --------------------------MANQLLST---VSCHDTLPEGYARPESDRPRLAE     31
O.sativa_Os04g49194              -------------------------MATTQLLST---VEHRETLPEGYARPESDRPRLAE     32
O.sativa_Os10g39140              --------------------MAAEAEQQHQLLST----AVHDTMPGKYVRPESQRPRLDL     36
S.bicolor_Sb01g030560            --------------------------MAEQLLST----AVHDTLPGSYVRPESQRPRLAE     30
ZmFNSI-1/ZmDMR6                  --------------------------MAEHLLST----AVHDTLPGSYVRPEPERPRLAE     30
B.distachyon_Bradi1g77040        --------------------------MAEQLIST----CAHETLPESYVRDAAERPRLDE     30
O.sativa_Os03g03034              --------------------------MADQLIST----ADHDTLPGNYVRPEAQRPRLAD     30
S.italica_Si036512m.g            --------------------------MADQLIST----AVHEELPENYVRPEAQRPRLHE     30
S.bicolor_Sb01g049030            --------------------------MAEQLIST----AEHDTLPDSYVRPETQRPRLRE     30
Z.mays_GRMZM2G475380             --------------------------MAEHLIST----ADHDRLPDSYVRPETQRPRLRE     30
A.coerulea_v1.008143m.g          -------------------------MESSNVLLT---GTRYSNLPENYVRSVSDRPRLSE     32
E.salsugineum_TP2g22820          --------------------------MAAKLIST---GFRHTTLPENYVRPHSDRPRLSE     31
C.rubella_006g21660              --------------------------MAAKLIST---GLRHTTLPGNYVRPISDRPRLSE     31
AtDMR6                           --------------------------MAAKLIST---GFRHTTLPENYVRPISDRPRLSE     31
AlDMR6                           --------------------------MAATLIST---GLRHTTLPENYVRPISDRPRLSE     31
C.sativus_Cucsa.273300           ----------------------MSSVMEIQLLCS---GGRHEKLPEKYERPESDRPRLSE     35
SlDMR6                           --------------------------METKVISS---GINHSTLPQSYIRPESDRPRLSE     31
M.guttatus_mgv1a009622m.g        --------------------------METKV-IS---GVHFSSLPASYIRPESDRPKLSE     30
VvDMR6.1                         --------------------------MESKVLST---GIRYLTLPQSYIRPEPERPRLSQ     31
C.papaya_16416436                --------------------------METKVLSS---GVRYENLPENYVRPESERPRLAE     31
VvDMR6.2                         --------------------------MDSKVLST---GIPFTTLPENYIRPESERPRLSE     31
R.communis_29866.t000021         --------------------------MDTKVLSS---GIRYSNLPESFIRPESERPRLSE     31
M.truncatula_Medtr3g122530       --------------------------MDTKVLSS---GIHYSKLPESYIRPESDRPCLSQ     31
G.max_Glyma04g227900             --------------------------MDTKVLSS---GVQYSNLPESYIRPESERPRLSE     31
G.max_Glyma06g137000             --------------------------MDIKVLSS---GVQYSNLPESYIRPESERPRLSE     31
G.max_Glyma06g14190              --------------------------MDIKVLSS---GVQYSNLPESYIRPESERPRLSE     31
E.grandis_v1_0.018520m.g         --------------------------MEAKVISG---GTRFTSLPRSYVRPESERPRLSE     31
E.grandis_v1_0.018494m.g         --------------------------MEAKVISG---GTRFTSLPRSYVRPESERPRLFE     31
E.grandis_v1_0.018496m.g         --------------------------MEAKVISG---GTRFTRLPRSYVRPESERPRLSE     31
P.persica_ppa008269m.g           --------------------------METKVLSS---GFKYENLPEGYVRPESERPRLSE     31
C.clementina_0.9_015118m.g       --------------------------MDTKVLSS---GIRYTNLPEGYVRPESERPNLSE     31
P.persica_ppa022381m.g           ---------------------------MAAKLLSD-LASGVTCVPSNYVRPVHDRPSLDQ     32
P.persica_ppa019415m.g           ---------------------------MAAKLLSD-LASGVTCVPSNYVRPVHDRPSLDQ     32
C.clementina_0.9_032642m.g       -----------------------MAAATTKLLLSD-LASTVKSVPSNYIRPISDRPNLTE     36
C.clementina_0.9_012078m.g       -----------------------MAAATTKLLLSD-LASTVKSVPSNYIRPISDRPNLTE     36
DLO
B.distachyon_Bradi5g19250        -----------------------MATAIAKPLLSD-LVAESGTVPSSHIRPVGDRPDLAD     36
O.sativa_Os04g49210              -----------------------MAPAIAKPLLSD-LVAQSGQVPSSHIRPVGDRPDLDN     36
S.italica_Si010491m.g            -----------------------MAPTIAKPLLSD-LVAQIGQVPLSHVRPVGDRPDLAN     36
```

```
S.bicolor_Sb06g026350            -----------------------MAPAISKPLLSD-LVAQIGKVPSSHIRPVGDRPDLAN    36
Z.mays_GRMZM2G050234             -----------------------MAPAISKPLLTD-LVAQIGKVPSSHIRPVGDRPDLAN    36
G.max_Glyma03g42250              -------------------------MAEKLVLVSD-MASTMKQVPSNFIRPLGDRPNLQG    34
G.max_Glyma16g01990              -------------------------MATTKPLLTD-LASTVDRVPSNFIRPIGDRPNLQQ    34
G.max_Glyma07g05420              -------------------------MAATKPLLTD-LASTIDRVPSNFIRPIGDRPKLHQ    34
VvDLO1                           -------------------------MANAKLLLSD-LASSIDCVPSRYVRPVNDRPNLDE    34
R.communis_30076.t000026         -------------------------MAIAKPLLSD-LVSTITSVPSNFIRPLSDRPNFNE    34
AlDLO1                           ----------------------MATSATSKLLVSD-FDSSVSHIPSNYVRPILDRPNLSE    37
AtDLO1                           ----------------------MATSAISKLLVSD-FASS-VHIPSNYVRPISDRPNLSE    36
AlDLO2                           ----------------------MAASATSKLLVSD-IASVVDHVPSNYVRPVSDRPNMSE    37
AtDLO2                           -------------------------MAASKLLVSD-IASVVDHVPSNYVRPVSDRPKMSE    34
C.sativus_Cucsa.193360           ----------------------MSASGHTKLLVTD-LAATVQQVPSRYVRPISDRPNSSD    37
C.papaya_evm.TU.sup_37.106       -------------------------MAATKLLLAD-LASNLKQVPAKYIQPISDRPNLAD    34
E.grandis_v1_0.017927m.g         -----------------------MATAATKLLLID-RVPGINRVPNSYIRPQADRPNLTE    36
E.grandis_v1_0.017897m.g         -----------------------MATAATKLLLID-RVPGINRVPNSYIRPEADRPNLTE    36
E.grandis_v1_0.047536m.g         MLTSAAQINTMTSYSALGRRFGENEKTAAKLLLTD-LASGIDRVPDNYIRSEADRPNLTE    59
E.grandis_v1_0.017895m.g         -----------------------MAPAAAKVLLTD-LASGIDRVPDNYIRSEADRPNLTE    36
E.grandis_v1_0.017876m.g         -----------------------MAPAAAKVLLTD-LASGIDRVPDNYIRSEADRPNLTD    36
E.grandis_v1_0.015926m.g         -----------------------MAPAAAKVLLTD-LASGIDRVPDNYIRSEADRPNLTE    36
VvDLO2 (V2D)                     -----------------------MVPSTTKLLLTD-MVLGVDHVPSNYVRPPSERPNFKD    36
C.clementina_0.9_014263m.g       --------------------MSAAATTATKLLLSD-LAPTLTNVPSDYIRPISDRPSLTD    39
P.persica_ppa008100m.g           ------------------------MATATKLLLTD-LMSGVNHVPSNYVRPISDRPNLSD    35
P.persica_ppa008091m.g           ------------------------MATATKLLLTD-LMSGVNHVPSNYVRPISDRPNLSD    35
R.communis_30068.t000102         ------------------------MAS-TKLLLADL-ASGVRHVPSKYIRPVSDRPNLSD    34
M.esculenta_4.1_029834m.g        ------------------------MAF-SKPLLADLSSLGVKNVPSSYIRPISDRPNLSD    35
                                                                            :
```

```
DMR6
B.distachyon_Bradi5g19240       VA--TDSNIPLIDLAS--P-DKLRVIAEIDRACRTYGFFQVINHGISEELLEKVMAVGLE     86
O.sativa_Os04g49194             VA--TDSNIPLIDLAS--P-DKPRVIAEIAQACRTYGFFQVTNHGIAEELLEKVMAVALE     87
O.sativa_Os10g39140             VV--SDARIPVVDLAS--P-DRAAVVSAVGDACRTHGFFQVVNHGIDAALIASVMEVGRE     91
S.bicolor_Sb01g030560           VV--TGARIPVVDLGS--P-DRAAVVAAIGDACRSHGFFQVLNHGVHADLVAAVMAVGRA     85
ZmFNSI-1/ZmDMR6                 VV--TGARIPVVDLGS--P-DRGAVVAAVGDACRSHGFFQVVNHGIHAALVAAVMAAGRG     85
B.distachyon_Bradi1g77040       VV--PDAHIPVVDLAH--P-DRAAIVSQIGAACRSHGFFQVLNHGLPAELMEAAMAVAHE     85
O.sativa_Os03g03034             VL--SDASIPVVDLAN--P-DRAKLVSQVGAACRSHGFFQVLNHGVPVELTLSVLAVAHD     85
S.italica_Si036512m.g           VV--SDAQIPVVDLAD--P-DPAAVVASIGEACTTHGFFQVLNHGVPVELMVAMLAVAYE     85
S.bicolor_Sb01g049030           VV--PDAEIPVVDLAV--P-DRAAVVARVAEACRTHGFFQVVNHGVAEELTAAMLAVAYE     85
Z.mays_GRMZM2G475380            VV--PDAEIPVVDLAD--P-DREAVVARVAEACRTHGFFQLLNHGVPEQLTAAMMSVAYE     85
A.coerulea_v1.008143m.g         VK--DCENVPVIDLSV--A-DESLLAQQIGNACKSHGFFQVINHGVNSELVEKMMEISHE     87
E.salsugineum_TP2g22820         VS--QLEDFPLIDLSS--T-DRSRLLQQIHQACARFGFFQVINHGVSKERIDEMVSVANE     86
C.rubella_006g21660             VS--QLEDFPLIDLSS--S-DRSLLVQQTHQACARFGFFQVTNHGVSKQVIDDMVSVAHE     86
AtDMR6                          VS--QLEDFPLIDLSS--T-DRSFLIQQIHQACARFGFFQVINHGVNKQIIDEMVSVARE     86
AlDMR6                          VS--QLEDFPLIDISS--T-DRSVLVQQIHQACARFGFFQVINHGVSKQLIDEMVSVAHE     86
C.sativus_Cucsa.273300          VC--CWDKVPIIDLGC--E-EREMIVKQVEEACKSYGFFQVINHGVRKELVEKVIEVGKQ     90
SlDMR6                          VV--DCENVPIIDLSC--G-DQAQIIRQIGEACQTYGFFQVINHGVPKEVVEKMLGVAGE     86
M.guttatus_mgv1a009622m.g       IE--EFDNVPVIDLGC--E-DHNLIVKQIGDACREYGFFQVINHGVSKALVDNILCVAHE     85
VvDMR6.1                        VS--ECKHVPIIDLGK--DVNRAQLIQHIADACRLYGFFQVINHGVAAEMMEKMLEVADE     87
C.papaya_16416436               VS--ACQNVPVVDLGC--D-DRTQIIQQISDACKDFGFFQVINHGVSEETRDGMIGVAKE     86
VvDMR6.2 (E53G)                 IA--DCENVPIIDLSC--D-DRAQIIEQLADACSRYGFFQVINHGVSAEAIEKMLHVANE     86
R.communis_29866.t000021        VL--ACDNVPIVDLGC--E-DGAQVVQQIGYACSNYGFFQVINHKVPDEVVADMLLVASE     86
M.truncatula_Medtr3g122530      VS--EFENVPIIDLGS--H-NRTQIVQQIGEACSSYGFFQVVNHGVPLEELKKTAEVAYD     86
G.max_Glyma04g227900            VS--ECEDVPIIDLGC--Q-NRAQIVHQIGEACRNYGFFQVINHGVALEAAKEMAEVAHG     86
G.max_Glyma06g137000            VS--ECEDVPIIDLGS--Q-NRAQIVHQIGEACRNYGFFQVINHGVALEAAKEMEEVAHG     86
G.max_Glyma06g14190             VS--ECEDVPIIDLGS--Q-NRAQIVHQIGEACRNYGFFQVINHGVALEAAKEMEEVAHG     86
E.grandis_v1_0.018520m.g        VS--AFEHVPIIDLGC--N-DRSQVVRQVGDACRVYGFFQVINHGVSTEAVERMQEVAAE     86
E.grandis_v1_0.018494m.g        VS--AFEHVPIIDLGC--N-DRSRVVRQVGDACRVYGFFQVINHGVSTEAVERMQEVAAE     86
E.grandis_v1_0.018496m.g        VS--AFEHVPIIDLGC--N-DRSRVVHQVGDACRVYGFFQVINHGVSTEAVERMQEVAAE     86
P.persica_ppa008269m.g          VS--ECKNIPVINLAS--E-NRAETVQQVGDACKSYGFFQVINHGVSTEAVEKMLGTATE     86
C.clementina_0.9_015118m.g      VS--ECENVPVIDLAC--D-DRSLIVQQVADACKNYGFFQAINHEVPLETVERVIEVAKE     86
P.persica_ppa022381m.g          VQPS-DHSIPLINLRGFDGSRRHEIINQIGLACQNYGFFQVQNHAIEEVVIDNMLKVARE     91
P.persica_ppa019415m.g          VQPS-DHSIPLINLHGFDGSRRHEIINQIGLACQNYGFFQVQNHAIEEAVIDNMLKVARE     91
C.clementina_0.9_032642m.g      -VQISDGSIPLIDLQVLDGPRRLDIIKQIGQACQHDGVFQVKNHGIPETIINSMLSITRA     95
C.clementina_0.9_012078m.g      -VQISDGSIPLVDLQVLNGPSRLDTIKQIGQACQHDGFFQVRNHGIPETIINNMLTIARA     95
DLO
B.distachyon_Bradi5g19250       VDHESGAGIPLIDLKHLDGPERRRVVEAIGSACETDGFFMVTNHGIPEAVVEGMLRVAKE     96
O.sativa_Os04g49210             VDHESGAGIPVIDLKQLDGPDRRKVVEAIGSACETDGFFMVKNHGIPEEVVEGMLRVARE     96
S.italica_Si010491m.g           VDNESGAGIPLIDLKKLNGPQRREVVEAIGRACGSDGFFMVTNHGIPAGVVEGMLRVARE     96
```

```
S.bicolor_Sb06g026350           VDNESGAGIPLIDLKMLNGPERRKVVEAIGRACESDGFFMVTNHGIPAAVVEGMLRVARE   96
Z.mays_GRMZM2G050234            VDNESGAGIPLIDLKKLNGPERRKVVEAIGKACESDGFFMVTNHGIPAAVVEGMLRVARE   96
G.max_Glyma03g42250             VVQSSDVCIPLIDLQDLHGPNRSHIIQQIDQACQNYGFFQVTNHGVPEGVIEKIMKVTRE   94
G.max_Glyma16g01990             LHSS-IASIPIIDLQGLGGSNHSQIIQNIAHACQNYGFFQIVNHGIPEEVVSKMVNVSKE   93
G.max_Glyma07g05420             LHSS-LASIPIIDLQGLGGSNHSQIIQNIAHACQTYGFFQIVNHGIQEEVVSKMVNVSKE   93
VvDLO1 (H52L)                   VQSSLDGSIPLIDLQDLHGPSRSHVIKQIAEACQIDGFFRVKNHGIPESVIHGMLSITKE   94
R.communis_30076.t000026        VIQTSDCSIPLIDLQGLDGPLRSTLVKEIGQACQGYGFFQVKNHGIPEDVIDKMLSVSRE   94
AlDLO1                          V-ESSSDSIPLIDLRELHGPNRAEVVQQLDSACSTYGFFQIKNHGVPDTTVDKMLTVARE   96
AtDLO1                          V-ESSGDSIPLIDLRDLHGPNRAVIVQQLASACSTYGFFQIKNHGVPDTTVNKMQTVARE   95
AlDLO2                          V-ETFGDSIPLIDLQDLHGPNRANIINQFAHACSSYGFFQIKNHGVPEEIIKQMMNVGRE   96
AtDLO2                          V-QTSGDSIPLIDLHDLHGPNRADIINQFAHACSSCGFFQIKNHGVPEETIKKMMNAARE   93
C.sativus_Cucsa.193360          VRPSNTYSFSVIDLHALDGPSRPDVIYQIRRACERDGFFLVKNHGVPEEMINGVMRITRE   97
C.papaya_evm.TU.sup_37.106      VEISELSSIPLIDLEGLDGPRRSEIINQIAQACELHGFFQVKNHGVPEEMINGILRLARE   94
E.grandis_v1_0.017927m.g        VEASDASTIPLIDLQGLFGPNRDDIIRQIGKACQRDGFFQIKNHGIPEEVVQAIMNIAGE   96
E.grandis_v1_0.017897m.g        VEASDASTIPLIDLQGLFGPNRDDIIRLIGEACQQDGFFQIKNHGIPEEVVQAIMNIAGE   96
E.grandis_v1_0.047536m.g        VEASDASYIPLVDLQGLSGPNRDDIIRQIGRACQQDGFFQIKNHGIPEEKVQAIMNIARE   119
E.grandis_v1_0.017895m.g        VEASDASSIPLVDLQGLSGPNRDDIIRQIGRACQQDGFFQIKNHGIPEEKVQAIMNIARE   96
E.grandis_v1_0.017876m.g        VEASDASSIPLVDLQGLSGPNRDDIIRQIGRACQQDGFFQIKNHGIPEEKVQAIMNIARE   96
E.grandis_v1_0.015926m.g        VEASDASSIPLVDLQGLSGPNRDDIIRQIGRACQQDGFFQIKNHGIPEEKVQAIMNIARE   96
VvDLO2                          -VQASDVSIPLIDLQDLQGPGRPDVVKQIGQACQHSGFFQIQNHGVSETMISNILRLARD   95
C.clementina_0.9_014263m.g      QTHISDGSIPLIDLQGLNGPRRSDIIKQIGQACQHCGFFQVKNHGISEAMINNMLSIART   99
P.persica_ppa008100m.g          -VQISDASIPLIDLKDLHGHNHSNIIKQIGLACQTDGFFQVKNHGVPEEMIKDMLSIARE   94
P.persica_ppa008091m.g          -VQISDASIPLIDLKDLHGHNHSNIIKQIGLACQTDGFFQVKNHGVPEEMIKDMLSIARE   94
R.communis_30068.t000102        -VHKSDGSIRLIDLKGLRSPNRALVIKQIGQACQTDGFFQVKNHGLPDEMINSIMRTARE   93
M.esculenta_4.1_029834m.g       -VEMSDAAIPLIDLQGLYGPNHSLVIAQIGRACQFDGFFQVKNHGIPEDVIDTILHTGTD   94
                                        . ::::                 **   *.*   ** :
```

**DMR6**		
B.distachyon_Bradi5g19240	FFRLPPEEKAKLYSDEPSKKIRLSTSFNVRKETVHNWRDYLRLHCHPLEEFVPDWPSNPE	146
O.sativa_Os04g49194	FFRLPPEEKEKLYSDEPSKKIRLSTSFNVRKETVHNWRDYLRLHCHPLEEFVPEWPSNPA	147
O.sativa_Os10g39140	FFRLPAEEKAKLYSDDPAKKIRLSTSFNVRKETVHNWRDYLRLHCYPLHQFVPDWPSNPP	151
S.bicolor_Sb01g030560	FFRLSPEEKAKLYSDDPARKIRLSTSFNVRKETVHNWRDYLRLHCHPLDEFVPDWPSNPP	145
ZmFNSI-1/ZmDMR6	FFRLPPEEKAKLYSDDPARKIRLSTSFNVRKETVHNWRDYLRLHCHPLDEFLPDWPSNPP	145
B.distachyon_Bradi1g77040	FFRLSPEEKAKLYSDDPAKKIRLSTSFNVRKETVHNWRDYLRLHCHPLEQFVPDWPSNPS	145
O.sativa_Os03g03034	FFRLPAEEKAKLYSDDPAKKIRLSTSFNVRKETVHNWRDYLRLHCYPLHRYLPDWPSNPP	145
S.italica_Si036512m.g	FFRLPAEEKAKLYSDDPGKKMRLSTSFNVRKETVHNWRDYLRLHCYPLEQYVPDWPANPP	145
S.bicolor_Sb01g049030	FFRLPAEEKAKLYSDDPGKKMRLSTSFNVRKETVHNWRDYLRLHCHPLEQYVPDWPDNPP	145
Z.mays_GRMZM2G475380	FFRLPAEEKAKLYSDDPAKKMRLSTSFNVRKETVHNWRDYLRLHCHPLEQYVPDWPDNPP	145
A.coerulea_v1.008143m.g	FFHLPLDVKMQFYSDDPTKTMRLSTSFNLKKESVHNWRDYLRLHCHPIEKYVQEWPSVPS	147
E.salsugineum_TP2g22820	FFSMTMEEKMKLYSDDPTKTTRLSTSFNVKKEEVNNWRDYLRLHCYPLHKYVHEWPSNPP	146
C.rubella_006g21660	FFSMSMEEKMKLYSDDPTRTTRLSTSFNVKKEEVNNWRDYLRLHCYPIHKYVHEWPSKPP	146
AtDMR6	FFSMSMEEKMKLYSDDPTKTTRLSTSFNVKKEEVNNWRDYLRLHCYPIHKYVNEWPSNPP	146
AlDMR6	FFSMSMEEKMKLYSDDPTKTTRLSTSFNVKKEEVNNWRDYLRLHCYPIHKYVHEWPSNPP	146
C.sativus_Cucsa.273300	FFELPMEEKLKFYSDDPSKTVRLSTSFNVRKEQFRNWRDYLRLHCYPLSNYTPHWPSNPP	150
SlDMR6	FFNLPVEEKLKLYSDDPSKTMRLSTSFNVKKETVHNWRDYLRLHCYPLEKYAPEWPSNPS	146
M.guttatus_mgv1a009622m.g	FFDLSVEEKMKLYSDDPTKTMRLSTSFNVKKETVHNWRDYLRLHCYPLEKYVPEWPSNPS	145
**VvDMR6.1 (Y89H)**	FYRLPVEEKMKLYSDDPTKTMRLSTSFNVNKEKVHNWRDYLRLHCYPLDQYTPEWPSNPP	147
C.papaya_16416436	FFSLPMEEKMKIYSDDPAKTTRLSTSFNVKKEKVHNWRDYLRLHCHPLHKYMPEWPSSPP	146
**VvDMR6.2**	FFQLPVEEKMKLYSDDPSKTMRLSTSFNVKKEKVHNWRDYLRLHCHPLEQYMPEWPSNPP	146
R.communis_29866.t000021	FFKLPLEEKLKIYSDDPTKTMRLSTSFNMKKEKVHNWRDYLRLHCYPLDKYISEWPSDPP	146
M.truncatula_Medtr3g122530	FFKLPVEEKMKLYSDDPTKTMRLSTSFNVNKEEVHNWRDYLRLHCYPLDNYVPEWPSNPP	146
G.max_Glyma04g227900	FFKLPVEEKLKLYSEDPSKTMRLSTSFNVKKETVHNWRDYLRLHCYPLDKYAPEWPSNPP	146
G.max_Glyma06g137000	FFKLPVEEKLKLYSEDTSKTMRLSTSFNVKKETVRNWRDYLRLHCYPLEKYAPEWPSNPP	146
G.max_Glyma06g14190	FFKLPVEEKLKLYSEDTSKTMRLSTSFNVKKETVRNWRDYLRLHCYPLEKYAPEWPSNPP	146
E.grandis_v1_0.018520m.g	FFRLPVEEKMKLYSEDPTKTMRLSTSFNVKKEKVHNWRDYLRLHCHPLEKYMEEWPANPP	146
E.grandis_v1_0.018494m.g	FFRLPEEEKMKLYSEDPTKTMRLSTSFNVKKEKVHNWRDYLRLHCHPLEKYMEEWPANPP	146
E.grandis_v1_0.018496m.g	FFRLPVEEKMKLYSEDPTKTMRLSTSFNVKKEKVHNWRDYLRLHCHPLEKYMEEWPANPP	146
P.persica_ppa008269m.g	FFSLPVEEKMKLYSDDPSKTMRLSTSFNVKKEKVHNWRDYLRLHCYPLEKYVPEWPSNPS	146
C.clementina_0.9_015118m.g	FFNLPVEEKLKLYSDDPSKTMRLSTSFNVNKEKVHNWRDYLRLHCYPLDKYVPEWPSNPS	146
P.persica_ppa022381m.g	FFHLPENERLKCFSDDPLKTTRLSTSFNVKTEKVSSWRDYLRLHCYPLEDYMHEWPSNPP	151
P.persica_ppa019415m.g	FFHLPESERLKCFSEDPLKTTRLSTSFNVKTEEVSSWRDYLRLHCYPLEDYMHEWPSNPP	151
C.clementina_0.9_032642m.g	FFKLPESERLKSYSDDPSKSTRLSTSFNVNTEKVSNWRDYLRLHCYPLQDYIHEWPSNPP	155
C.clementina_0.9_012078m.g	FFKLPESERLKSYSDDPSKSTRLSTSFNVNTEKISNWRDYLRLHCYPLQDYMHEWPSNPP	155
**DLO**		
B.distachyon_Bradi5g19250	FFHLPESERLKCYSDDPKKAIRLSTSFNVRTEKVSNWRDFLRLHCYPLQSFIDQWPSNPP	156
O.sativa_Os04g49210	FFHMPESERLKCYSDDPKKAIRLSTSFNVRTEKVSNWRDFLRLHCYPLESFIDQWPSNPP	156
S.italica_Si010491m.g	FFHMPESERLKCYSDDPKKAIRLSTSFNVRTEKVSNWRDFLRLHCYPLESFIEQWPSNPP	156

```
S.bicolor_Sb06g026350          FFHLPESERLKCYSDDPKKAIRLSTSFNVRTEKVNNWRDFLRLHCYPLESFVDQWPSNPP    156
Z.mays_GRMZM2G050234           FFHLPESERLKCYSDDPNKAIRLSTSFNVRTEKVSNWRDFLRLHCYPLQSFVDQWPSNPP    156
G.max_Glyma03g42250            FFGLPESEKLKSYSTDPFKASRLSTSFNVNSEKVSSWRDFLRLHCHPIEDYIKEWPSNPP    154
G.max_Glyma16g01990            FFGLPESERLKNYSDDPTKTTRLSTSFNVKTEKVSNWRDFLRLHCHPLEDYIQEWPGNPP    153
G.max_Glyma07g05420            FFGLPESERLKNFSDDPSKTTRLSTSFNVKTEKVSNWRDFLRLHCHPLEDYIQEWPGNPP    153
VvDLO1                         FFHLPESERLKNYSDDPLKTMRLSTSFNVKTEQVSNWRDFLRLYCYPLEDYIQEWPSNPP    154
R.communis_30076.t000026       FFHLPESERMKNYSDDPMMRTRLSTSFNVRTEKTSNWRDFLRLHCYPLDDYMQEWPTNPP    154
AlDLO1                         FFHQPESERVKHYSADPTKTTRVSTSFNIGADKILNWRDFLRLHCFPIEDFIEEWPSSPN    156
AtDLO1                         FFHQPESERVKHYSADPTKTTRLSTSFNVGADKVLNWRDFLRLHCFPIEDFIEEWPSSPI    155
AlDLO2                         FFHQSESERVKHYSADTKKTTRLSTSFNVSKEKVSNWRDFLRLHCYPIEDFIHEWPSTPV    156
AtDLO2                         FFRQSESERVKHYSADTKKTTRLSTSFNVSKEKVSNWRDFLRLHCYPIEDFINEWPSTPI    153
C.sativus_Cucsa.193360         FFRLPESERLKSYSDDPTKKTRLSTSFNVKTEKVANWRDFLRLHCYPLHLYVDEWPSNPP    157
C.papaya_evm.TU.sup_37.106     FFRLPESQRLENYSDDPAKTTRLSTSFNVKTEKFSNWRDFLRLHCYPVQDYIHEWPTNPP    154
E.grandis_v1_0.017927m.g       FFRLPESERLKNYSDDPSKSTRLSTSFNIKTEKVSNWRDFLRLHCYPLEEYMHEWPTNPP    156
E.grandis_v1_0.017897m.g       FFRLPESERLKNYSDDPSKSTRLSTSFNIKTEKVSNWRDFLRLHCYPLEEYMHEWPTNPP    156
E.grandis_v1_0.047536m.g       FFHLPESERLKNYSDDPTKSTRLSTSFNLKTEKVSNWRDFLRLHCYPLEEYMHEWPTNPP    179
E.grandis_v1_0.017895m.g       FFHLPESERLKNYSDDPTKSTRLSTSFNLKTETVSNWRDFLRLHCYPLEEYMQEWPTNPP    156
E.grandis_v1_0.017876m.g       FFQLPESERLKNYSDDPTKSTRLSTSFNLKTETVSNWRDFLRLHCYPLEEYMHEWPTNPP    156
E.grandis_v1_0.015926m.g       FFRLPESERLKNYSDDPTKSTRLSTSFNLKTEKVSNWRDFLRLHCYPLEEYMHEWPTNPP    156
VvDLO2                         FFQLPESERLKNYSDNPSNPVRLSTSFNVKTEKVANWRDFLRLHCYPLEDYVHQWPSNPP    155
C.clementina_0.9_014263m.g     FFKLPESERLKIYSDDPSKPTRLSTSFNVKTEKVSNWRDFLRLHCYPLQDYVHDWPLNPP    159
P.persica_ppa008100m.g         FFKLPESERLKMYSDDPSKTTRLSTSFNVRTEKLSNWRDFLRLHCYPLEDYVQEWPNNPP    154
P.persica_ppa008091m.g         FFKLPESERLKMYSDDPSKTTRLSTSFNVRTEKLSNWRDFLRLHCYPLEDYVQEWPNNPP    154
R.communis_30068.t000102       FFKLPESERLKCYSNDPTKTTRLSTSFNVKTEKVSNWRDFLRLHCYPLADYIQEWPCNPP    153
M.esculenta_4.1_029834m.g      FFKLPESERLKSYSDDPAKTTRLSTSFNVKTEKFSNWRDFLRLHCYPVEDYIQEWPSNPP    154
                               *:    . : : :* :     *:*****:  :   .***:***:*.*:  :  .**  *
```

```
DMR6
B.distachyon_Bradi5g19240        AF-KEIISTYCREVRLLGLRLMGAISLSLGLDENYVEN-VLG----EQEQHMAVNYYPRC    200
O.sativa_Os04g49194              QF-KEIMSTYCREVRQLGLRLLGAISVSLGLEEDYIEK-VLG----EQEQHMAVNYYPRC    201
O.sativa_Os10g39140              SF-KEIIGTYCTEVRELGFRLYEAISESLGLEGGYMRE-TLG----EQEQHMAVNYYPQC    205
S.bicolor_Sb01g030560            DF-KDTMSTYCKEVRELGFRLYAAISESLGLEASYMKE-TLG----EQEQHMAVNFYPPC    199
ZmFNSI-1/ZmDMR6                  DF-KETMGTYCKEVRELGFRLYAAISESLGLEASYMKE-ALG----EQEQHMAVNFYPPC    199
B.distachyon_Bradi1g77040        AF-REVMSTYCKEIRELGFRLYAAISESLGLEEDYMKK-VLG----EQEQHMAVNFYPKC    199
O.sativa_Os03g03034              SF-REIISTYCKEVRELGFRLYGAISESLGLEQDYIKK-VLG----EQEQHMAVNFYPKC    199
S.italica_Si036512m.g            SF-REIVSAYCREVRALGFRLYEAISASLGLEDDYVKR-TLG----EQEQHMAVNFYPRC    199
S.bicolor_Sb01g049030            SF-RETVSAYCREVRALGFRLYGAISEGLDLDGVYIKE-TLG----EQEQHMAVNFYPRC    199
Z.mays_GRMZM2G475380             SF-RRTVSAYCSAVRELGFRLYVAISEGLGLDGAYIKE-ALG----EQEQHMAVNFYPRC    199
A.coerulea_v1.008143m.g          TF-KDVVATYCKEVRKLGLRLLGSISLSLGLEEDYIEK-VLG----DQGQHMAVNYYPPC    201
E.salsugineum_TP2g22820          SF-KEIVSKYSRQVRDVGCTIEELISESLGLEKDYMKK-VLG----EQGQHMAINYYPPC    200
C.rubella_006g21660              SF-KEVVSKYSREVREVGFNIEELISESLGLEKDYLKK-VLG----EQGQHMAVNYYPPC    200
AtDMR6                           SF-KEIVSKYSREVREVGFKIEELISESLGLEKDYMKK-VLG----EQGQHMAVNYYPPC    200
AlDMR6                           SF-KEIVSKYSREVREVGFKIEELISESLGLEKDYMKK-VLG----EQGQHMAVNYYPPC    200
C.sativus_Cucsa.273300           SF-REIVSSYCNEVRKVGYRIEELISESLGLEKEYIRK-KLG----EQGQHMAINYYPPC    188
SlDMR6                           SF-REIVSRYCREIRQLGFRLEEAIAESLGLDKECIKD-VLG----EQGQHMAINYYPPC    200
M.guttatus_mgv1a009622m.g        SF-KDVVSTYCAEIRQLGLRLQEDISESLGLDKDNLKN-VLG----DQGQHMAVNYYPAC    199
VvDMR6.1                         SF-KEIVSSYCKEVRELGFRLQEMISESLGLEKDHIKN-VFG----EQGQHMAVNYYPPC    201
C.papaya_16416436                SF-KEVVSKYSIEVRELGLRIEELISESLGLDKDLIRN-VVG----EQGQHMAVNYYPPC    200
VvDMR6.2                         EF-KDTVSNYCVEVRQLGHRLEEAIGESLGLEKDYIRN-TLG----EQGQHMAVNYYPPC    200
R.communis_29866.t000021         LF-KEIVSRYCIEVRKLGFRLQELISESLGLPKDHIRN-VLG----EQGQHMAVNYYPPC    200
M.truncatula_Medtr3g122530       SF-KETVANYCKEVRELGLRIEEYISESLGLEKDYLRN-ALG----EQGQHMAVNYYPPC    200
G.max_Glyma04g227900             SF-KETVTEYCTLVRELGLRIQEYISESLGLEKDYIKN-VLG----EQGQHMAVNYYPPC    200
G.max_Glyma06g137000             SF-KETVTEYCTIIRELGLRIQEYISESLGLEKDYIKN-VLG----EQGQHMAVNYYPPC    200
G.max_Glyma06g14190              SF-KETVTEYCTIIRELGLRIQEYISESLGLEKDYIKN-VLG----EQGQHMAVNYYPPC    200
E.grandis_v1_0.018520m.g         TF-KEFVSNYCREVRRLGYRLEELISESLGLEKDAVRN-ILG----EQGQHMAVNFYPPC    200
E.grandis_v1_0.018494m.g         TF-KEFVSNYCREVRRLGYRLEELISESLGLEKDAVRN-ILG----EQGQHMAVNFYPPC    200
E.grandis_v1_0.018496m.g         TF-KEFVSNYCREVRRLGYRLEELISESLGLEKDAVRN-ILG----EQGQHMAVNFYPPC    200
P.persica_ppa008269m.g           SF-KDIVSKYSEEVRELGFRLQELISESLGLEKDYIKS-TLG----EQGQHMAVNFYPPC    200
C.clementina_0.9_015118m.g       TF-KEFVSTYCSEVRGLGYRVLELISESLGLEKDYIKK-VLG----EQGQHMAVNFYPPC    200
P.persica_ppa022381m.g           SF-REDVAEYCRNVKGLAERLLEAISESLGLEKDYMNR-ALG----KHGQHMAINYYPPC    205
P.persica_ppa019415m.g           SF-REDVAEYCRNVKGLAERLLEAISESLGLEKDYMNR-ALG----KHGQHMAINYYPPC    205
C.clementina_0.9_032642m.g       SF-RYN------YARGLVLRLLEAISESLGLQRDYIDK-ALG----KHRQHMALNYYPHC    203
C.clementina_0.9_012078m.g       SV-REVVAEYCTSVRGLVLRLLEAISESLGLQRDFIDK-ALG----KHGQHMALNYYPPC    209
DLO
B.distachyon_Bradi5g19250        AF-REVVGAYSTEARALALRLLEAISESLGLERRHMVT-AMG----GHAQHMAVNYYPPC    210
O.sativa_Os04g49210              SF-RQVVGTYSREARALALRLLEAISESLGLERGHMVS-AMG----RQAQHMAVNYYPPC    210
S.italica_Si010491m.g            SF-REVVGTYATEARALALRLLEAISESLGLERSHMVA-AMG----RQAQHMAVNYYPPC    210
```

```
S.bicolor_Sb06g026350            SF-RQVVGTYATEARALALRLLEAISESLGLERSHMVR-AMG----RHAQHMAVNYYPPC    210
Z.mays_GRMZM2G050234             SF-RQVVGTYATEARALALRLLEAISESLGLERSHMVA-AMG----RHAQHMAVNYYPPC    210
G.max_Glyma03g42250              SLSREDVAEYCRKMRGVSLKLVEAISESLGLERDYINR-VVGGKKGQEQQHLAMNYYPAC    213
G.max_Glyma16g01990              SF-REDVAEYSRKMRGLSLKLLEAISESLGLEKDYIDK-ALG----KHGQHMAINYYPPC    207
G.max_Glyma07g05420              SF-REDVAEYSRKMRGLSLKLLEAISESLGLERDYIDK-ALG----KHGQHLAINYYPPC    207
VvDLO1                           SF-REVVAEYCKEARKLALLLLEAISESLGLERNHIDK-ALG----KHSQQMALNYYPPC    208
R.communis_30076.t000026         SF-REDVGEYCRNVRDLAVRLLEAISESLGLERDYINK-ALD----KHAQHLAVNYYPSC    208
AlDLO1                           SF-KEVTAEYATSVRALVLRLLEAISESLGLESDHISN-ILG----KHAQHMAFNYYPPC    210
AtDLO1                           SF-REVTAEYATSVRALVLRLLEAISESLGLESDHISN-ILG----KHAQHMAFNYYPPC    209
AlDLO2                           SF-REVTAEYATSVRALVLTLLEAISESLGLVKDRVSN-TLG----KHGQHMAINYYPPC    210
AtDLO2                           SF-REVTAEYATSVRALVLTLLEAISESLGLAKDRVSN-TIG----KHGQHMAINYYPRC    207
C.sativus_Cucsa.193360           SF-RKEVAEYCTTMRQLTLKLLEAISESLGLPKDSIAN-SIG----SHGQHMALNYYPPC    211
C.papaya_evm.TU.sup_37.106       FF-REDVAEYCSRIRGLVLKLVEAISESLGLGGDYINK-VLG----KHGQHMAFNYYPPC    208
E.grandis_v1_0.017927m.g         SF-RKEVGEYCTRVRELALKLLEAISESLGLEREYISK-NLG----KHGQHMAMNYYPPC    210
E.grandis_v1_0.017897m.g         SF-RKEVGEYCTRVRELALKLLEAISESLGLEREYISK-NLG----KHGQHMAMNYYPPC    210
E.grandis_v1_0.047536m.g         SF-RKEVGEYCTRVRELVFKLLEAISESLGLEREYISQ-NLG----EHGQHMAMNYYPPC    233
E.grandis_v1_0.017895m.g         SF-RKEVGEYCTRVRELVLKLLEAISESLGLEREYISQ-NLG----KHGQHMAMNYYPPC    210
E.grandis_v1_0.017876m.g         SF-RKEMGEYCTRVRELVLKLLEAISESLGLEREYISQ-NLG----KHGQHMAMNYYPPC    210
E.grandis_v1_0.015926m.g         SF-RKEVGEYCTRVRELVFKLLEAISESLGLEREYISQ-NLG----KHGQHMAMNYYPPC    210
VvDLO2                           SF-REDVAEYCTSIRALVLRLLETISESLGLEKNYVSG-VLG----KHGQHMAMNYYPPC    209
C.clementina_0.9_014263m.g       SF-REDVGDYCTSVRGLVLRLIQAISESLGLPSDYIDKEALG----KHGQHMALNYYPPC    214
P.persica_ppa008100m.g           SF-REQVGEYCTTVRGLVLRLLGAISESLGLEKNYIVE-ALG----KQGQHMALNYYPPC    208
P.persica_ppa008091m.g           SF-REQVGEYCTTVRGLVLRLLGAISESLGLEKNYIVE-ALG----KQGQHMALNYYPPC    208
R.communis_30068.t000102         LF-RKNVSEYSTSVRRLVLTLLEAISESLGLKRDYIEK-TLS----KQGQHMAMNYYPPC    207
M.esculenta_4.1_029834m.g        SF-RKNVAEYCTRVRGLVLTLLEAISESLGLKSDYIDK-ALS----KHGQHMAINYYPPC    208
                                  .                  :   *. .*.*    :    .
```

```
DMR6
B.distachyon_Bradi5g19240      PEPDLTYGLPKHTDPNALTVLLQDPNVSGLQVLKDG-QWIAVDPRPNALVINLGDQLQ--    257
O.sativa_Os04g49194            PEPDLTYGLPKHTDPNALTILLPDPHVAGLQVLRDGDQWIVVNPRPNALVVNLGDQIQ--    259
O.sativa_Os10g39140            PEPELTYGLPAHTDPNALTILLMDDQVAGLQVLNDG-KWIAVNPQPGALVINIGDQLQ--    262
S.bicolor_Sb01g030560          PEPELTYGLPAHTDPNALTILLMDQDVAGLQVLHGG-KWVAVNPQPGALIINIGDQLQ--    256
ZmFNSI-1/ZmDMR6                PEPELTYGLPAHTDPNALTILLMDPDVAGLQVLHAG-QWVAVNPQPGALIINIGDQLQ--    256
B.distachyon_Bradi1g77040      PSPELTYGLPAHTDPNALTILLMDEQVAGLQVLKDG-QWIAVNPRPNALVVNLGDQLQ--    256
O.sativa_Os03g03034            PEPELTFGLPAHTDPNALTILLMDQQVAGLQVLKEG-RWIAVNPQPNALVINIGDQLQ--    256
S.italica_Si036512m.g          PAPELTYGLPAHTDPNALTILLMDQQVAGLQVLNDG-RWIAVNPRPNALVINIGDQLQ--    256
S.bicolor_Sb01g049030          PAPELTYGLPAHTDPNALTILLMDQQVAGLQVLKDG-RWIAVNPRPGALVVNLGDQLQ--    256
Z.mays_GRMZM2G475380           PAPELTYGLPAHTDPNALTILLMDQQVAGLQVLKDG-RWIAVNPRPGALVVNIGDQLQVT    258
A.coerulea_v1.008143m.g        PEPELTYGLPRHTDPNTITILLQGQEVAGLQVLHNG-KWVAVNPYPNAFVVNIGDQIQ--    258
E.salsugineum_TP2g22820        PEPELTYGLPAHTDPNVLTILLQDATVCGLQILIDG-HWFAVNPRPDAFVINIGDQLQ--    257
C.rubella_006g21660            PEPELTYGLPAHTDPNALTILLQDSTVCGLQILIDG-QWFAVNPHPNAFVINIGDQLQ--    257
AtDMR6                         PEPELTYGLPAHTDPNALTILLQDTTVCGLQILIDG-QWFAVNPHPDAFVINIGDQLQ--    257
AlDMR6                         PEPELTYGLPAHTDPNALTILLQDTTVCGLQILIDG-QWFAVNPHPDAFVINIGDQLQ--    257
C.sativus_Cucsa.273300         PQPELTYGLPGHTDPNALTILLQDLHVAGLQVLKDG-KWLAVNPHPNAFVINIGDQLQ--    245
SlDMR6                         PQPELTYGLPAHTDPNSLTILLQDLQVAGLQVLKDG-KWLAVKPQPDAFVINLGDQLQ--    257
M.guttatus_mgv1a009622m.g      PEPELTYGLPAHTDPNALTILLQDLQVAGLQVLKDG-KWLAIKPQPGAFVINIGDQLQ--    256
VvDMR6.1 (I253K)               PQPELTYGLPGHTDPNALTILLQDLRVAGLQVLKDG-TWLAIKPHPGAFVVNIGDQLQ--    258
C.papaya_16416436              PQPELTYGLPAHTDPNALTILLQDLQVSGLQVLKDG-KWVAVHPQPNAFVINIGDQLQ--    257
VvDMR6.2                       PEPELTYGLPAHTDPNALTILLQDSHVAGLQVLKDG-KWVAVKPHPGAFVVNIGDQLQ--    257
R.communis_29866.t000021       PQPDLTYGLPGHTDPNALTILLQDLQVAGLQVFKDG-KWLAVNPHPNAFVINLGDQLQ--    257
M.truncatula_Medtr3g122530     PQPELTYGLPGHTDPNALTILLQDLHVAGLQVLKDG-KWLAINPIPDAFVINIGDQLQ--    257
G.max_Glyma04g227900           PEPELTYGLPGHTDPNALTILLQDLQVCGLQVLKNG-KWLAVNPQPNAFVINIGDQLQ--    257
G.max_Glyma06g137000           PEPELTYGLPGHTDPNALTILLQDLQVAGLQVLKDG-KWLAVSPQPNAFVINIGDQLQ--    257
G.max_Glyma06g14190            PEPELTYGLPGHTDPNALTILLQDLQVAGLQVLKDG-KWLAVSPQPNAFVINIGDQLQ--    257
E.grandis_v1_0.018520m.g       PEPELTYGLPGHTDPNALTILLQDPHVAGLQVLKDG-KWVAIDPHPNAFVINIGDQLQ--    257
E.grandis_v1_0.018494m.g       PEPELTYGLPGHTDPNALTILLQDLHVAGLQVLKDG-KWVAIDPHPNAFVINIGDQLQ--    257
E.grandis_v1_0.018496m.g       PEPELTYGLPGHTDPNALTILLQDLHVAGLQVLKDG-KWVAIDPHPNAFVINIGDQLQ--    257
P.persica_ppa008269m.g         PQPELTYGLPGHTDPNALTILLQDLEVAGLQVLKDG-KWIAVNPHPNAFVINLGDQLQ--    257
C.clementina_0.9_015118m.g     PEPELTYGLPGHTDPNALTILLQDLEVAGLQVLKDD-KWVAVNPLPNAFVINIGDQLQ--    257
P.persica_ppa022381m.g         HQPELTYGLPGHADPNVVTLLLQD-DVAGLQVFNNG-RWVAVKPMPHTFIVNIGDQIQ--    261
P.persica_ppa019415m.g         HQPELTYGLPGHADPNVVTLLLQD-DVAGLQVFNNG-RWVAVKPMPHTFIVNIGDQIQ--    261
C.clementina_0.9_032642m.g     PQPDLTYGLPGHIDPNLITVLLQD-DVPGLQVLRKG-KWLPVSPIPNTFIVKIGDQMQ--    259
C.clementina_0.9_012078m.g     PQPDLTYGLPGHTDPNLITVLLQD-DVPGLQVLRNG-KWLPVSPIPNTFIVNIGDQMQ--    265
DLO
B.distachyon_Bradi5g19250      PQPELTYGLPGHKDPNAVTLLLQD-GVSGLQVQRGG-RWVAVNPVPNALVINIGDQLQ--    266
O.sativa_Os04g49210            PQPELTYGLPGHKDPNAITLLLQD-GVSGLQVQRNG-RWVAVNPVPDALVINIGDQIQ--    266
S.italica_Si010491m.g          PQPELTYGLPGHKDPNAITLLLQD-GVSGLQVQRDG-RWVAVNPVPNALVINIGDQLQ--    266
```

```
S.bicolor_Sb06g026350           PQPELTYGLPGHKDPNAITLLLQD-GVSGLQVQRGG-RWVAVNPVPDALVINIGDQMQ--    266
Z.mays_GRMZM2G050234            PQPELTYGLPGHKDPNAITLLLQD-GVSGLQVQRGG-RWVAVNPVPNALVINIGDQMQ--    266
G.max_Glyma03g42250             PEPELTYGLPGHTDPTVITILLQD-EVPGLQVLKDG-KWVAVNPIPNTFVVNVGDQIQ--    269
G.max_Glyma16g01990             PEPELTYGLPAHADPNAITILLQN-QVPGLQVLHDG-KWLTVNPVPNTFIVNIADQIQ--    263
G.max_Glyma07g05420             PEPELTYGLPAHADPNAITILLQN-EVPGLQVLYDG-KWLTVNPVPNTFIVNIGDQIQ--    263
VvDLO1                          PQPELTFGLPGHADPNALTILLQD-DVPGLQVLKDG-KWVAIHPIPNTFIVNIGDQIQ--    264
R.communis_30076.t000026        PQPELTYGLPVHADPNVITILLQD-DVPGLQVLKDG-KWVAVSPVPHTFIVNIGDQIQ--    264
AlDLO1                          PEPELTYGLPGHKDPTVITVLLQD-QVSGLQVFKDN-KWVAVNPIPNTFIVNIGDQMQ--    266
AtDLO1                          PEPELTYGLPGHKDPTVITVLLQD-QVSGLQVFKDD-KWVAVSPIPNTFIVNIGDQMQ--    265
AlDLO2                          PQPELTYGLPGHKDANLITVLLQD-EVSGLQVFEDG-KWIAVNPIPNTFIVNLGDQMQ--    266
AtDLO2                          PQPELTYGLPGHKDANLITVLLQD-EVSGLQVFKDG-KWIAVNPVPNTFIVNLGDQMQ--    263
C.sativus_Cucsa.193360          PQPDLTYGLPCHTDPNLITLLLQD-QVPGLQVHRDG-AWVALNPIPNTFIINIGDQMQ--    267
C.papaya_evm.TU.sup_37.106      PEPELTYGLPGHTDPNLITVLLQD-DVPGLQVLRNG-KWVAVNPIPNTFIINIGDQMQ--    264
E.grandis_v1_0.017927m.g        PQPELTYGLPGHTDQNLITILLQD-EVPGLQVLRDG-KWIAVNPIPNTFIVNIGDQMQ--    266
E.grandis_v1_0.017897m.g        PQPELTYGLPGHTDRNLITILLQD-DVPGLQVLRDG-KWIAVNPIPNTFIVNIGDQMQ--    266
E.grandis_v1_0.047536m.g        PQPELTYGLPSHTDPNLITILLQD-DVPGLQVLRNG-KWVAVNPIPNTFIVNIGDQMQ--    289
E.grandis_v1_0.017895m.g        PQPELTYGLPGHTDPNLITILLQD-DVPGLQVLRNG-KWVAVNPIPNTFIVNIGDQMQ--    266
E.grandis_v1_0.017876m.g        PQPELTYGLPGHTDPNLITILLQD-DVPGLQVLRNG-KWVAVNPIPNTFIVNIGDQMQ--    266
E.grandis_v1_0.015926m.g        PQPELTYGLPGHTDPNLITILLQD-DVPGLQVLRNG-KWVAVNPIPNTFIVNIGDQMQ--    266
VvDLO2                          PQPELTYGLPGHTDCSLITVLLQD-DVPGLQVLRNG-KWVSVNPIPNSFIVNIGDHMQ--    265
C.clementina_0.9_014263m.g      PQPELTYGLPGHTDPNLITLLLQD-DVPGLQVLRDG-NWVPVNPIPSTFIVNIGDQMQ--    270
P.persica_ppa008100m.g          PEPELTYGLPGHTDCNLITILLQD-DVAGLQVLRNG-KWVAVNPIPNTFIVNIGDMMQ--    264
P.persica_ppa008091m.g          PEPELTYGLPGHTDCNLITILLQD-DVAGLQVLRNG-KWVAVNPIPNTFIVNIGDMMQ--    264
R.communis_30068.t000102        PQPELTYGLPGHTDPNLITILLQD-HVPGLQVLRNG-KWIAINPIPSTFIVNIGDQMQ--    263
M.esculenta_4.1_029834m.g       PQPELTYGLPEHSDPNLITILLQD-QVPGLQVLRNG-KWVAVDPIPNTFIVNIGDQMQ--    264
                                  *:**:*** * * . :*:** .  * ***:   .  *. : * * ::::::.* :*
```

**DMR6**		
B.distachyon_Bradi5g19240	----------------------------ALSNGAYKSVWHRAVVNAAQERMSVASFLCPC	289
O.sativa_Os04g49194	----------------------------ALSNDAYKSVWHRAVVNPVQERMSVASFMCPC	291
O.sativa_Os10g39140	----------------------------ALSNGKYRSVWHRAVVNSDRERMSVASFLCPC	294
S.bicolor_Sb01g030560	----------------------------ALSNGQYRSVWHRAVVNSDRERMSVASFLCPC	288
ZmFNSI-1/ZmDMR6	----------------------------ALSNGQYRSVWHRAVVNSDRERMSVASFLCPC	288
B.distachyon_Bradi1g77040	----------------------------ALSNGRYKSVWHRAVVNSDRPRMSIASFMCPC	288
O.sativa_Os03g03034	----------------------------ALSNGRYKSVWHRAVVNSDKARMSVASFLCPC	288
S.italica_Si036512m.g	----------------------------ALSNGRYKSVWHRAVVNSDRPRMSVASFLCPC	288
S.bicolor_Sb01g049030	----------------------------ALSNGRYKSVWHRAVVNSDRPRMSVASFLCPC	288
Z.mays_GRMZM2G475380	CSCLALPLFPPSHLLSAPGLHYCTAPAQALSNGRYRSVWHRAVVNADRPRMSVASFLCPC	318
A.coerulea_v1.008143m.g	----------------------------ALSNGNYASVWHRATVNTDRERISVASFLCPA	290
E.salsugineum_TP2g22820	----------------------------ALSNGVYKSVWHRAVTNTDKPRLSVASFLCPA	289
C.rubella_006g21660	----------------------------ALSNGVYKSVWHRAVTNTENPRLSVASFLCPA	289
AtDMR6	----------------------------ALSNGVYKSVWHRAVTNTENPRLSVASFLCPA	289
AlDMR6	----------------------------ALSNGVYKSVWHRAVTNTENPRLSVASFLCPA	289
C.sativus_Cucsa.273300	----------------------------ALSNGVYKSVWHRAVVNVDKPRLSVASFLCPC	277
SlDMR6	----------------------------AVSNGKYRSVWHRAIVNSDQARMSVASFLCPC	289
M.guttatus_mgv1a009622m.g	----------------------------ALSNGKYKSVWHRAVVNADKARLSVASFLCPC	288
**VvDMR6.1**	----------------------------AVSNGKYKSVWHRAVVNAESERLSVASFLCPC	290
C.papaya_16416436	----------------------------AVSNGKYKSVWHRAVVNSDKVRLSIASFLCPC	289
**VvDMR6.2**	----------------------------ALSNGKYRSVWHRATVNVGKARMSIASFLCPS	289
R.communis_29866.t000021	----------------------------ALSNGRYKSVWHRAIVNADRERMSIASFLCPC	289
M.truncatula_Medtr3g122530	----------------------------ALSNGLYKSVWHRAIVNAEKPRLSVASFLCPD	289
G.max_Glyma04g227900	----------------------------ALSNGLYKSVWHRAVVNVEKPRLSVASFLCPN	289
G.max_Glyma06g137000	----------------------------ALSNGLYKSVWHRAVVNVEKPRLSVASFLCPN	289
G.max_Glyma06g14190	----------------------------ALSNGLYKSVWHRAVVNVEKPRLSVASFLCPN	289
E.grandis_v1_0.018520m.g	----------------------------ALSNGRYKSVWHRAIVNADKPRMSIASFLCPS	289
E.grandis_v1_0.018494m.g	----------------------------ALSNGRYKSVWHRAIVNADKPRMSIASFLCPS	289
E.grandis_v1_0.018496m.g	----------------------------ALSNGRYKSVWHRAIVNADKPRMSIASFLCPS	289
P.persica_ppa008269m.g	----------------------------ALSNGIYRSVWHRAITNTDRARLSVASFLCPQ	289
C.clementina_0.9_015118m.g	----------------------------ALSNGRYKSVWHRAIVNAEKARMSVASFLCPN	289
P.persica_ppa022381m.g	----------------------------VVSNDRYKSVLHRAVVNCDKERISIPTFYCPS	293
P.persica_ppa019415m.g	----------------------------VVSNDRYKSVLHRAVVNCDKERISIPTFYCPS	293
C.clementina_0.9_032642m.g	----------------------------VLSNDRYKSVLHRALVNCDKERISIPTFYCPS	291
C.clementina_0.9_012078m.g	----------------------------VLSNDRYKSVLHRALVNCDRERISIPTFYCPS	297
**DLO**		
B.distachyon_Bradi5g19250	----------------------------ALSNDRYKSVLHRVIVNSESERISVPTFYCPS	298
O.sativa_Os04g49210	----------------------------ALSNDRYKSVLHRVIVNSESERISVPTFYCPS	298
S.italica_Si010491m.g	----------------------------ALSNDRYKSVLHRVIVNSESERISVPTFYCPS	298

```
S.bicolor_Sb06g026350           ----------------------------ALSNDRYKSVLHRVIVNSESERISVPTFYCPS    298
Z.mays_GRMZM2G050234            ----------------------------ALSNDRYKSVLHRVIVNSESERISVPTFYCPS    298
G.max_Glyma03g42250             ----------------------------VISNDKYKSVLHRAVVNCNKDRISIPTFYFPS    301
G.max_Glyma16g01990             ----------------------------VISNDRYKSVLHRALVNCEKERMSIPTFYCPS    295
G.max_Glyma07g05420             ----------------------------VISNDRYKSVLHRALVNCEKERMSIPTFYCPS    295
VvDLO1                          ----------------------------VLSNDCYKSAVHRAVVNCQKERISIPTFYCPS    296
R.communis_30076.t000026        ----------------------------VISNDRYKSVLHRAVVNSNKERISIPTFYCPS    296
AlDLO1                          ----------------------------VISNDKYKSVLHRAVVNTEKERLSIPTFYFPS    298
AtDLO1                          ----------------------------VISNDKYKSVLHRAVVNTENERLSIPTFYFPS    297
AlDLO2                          ----------------------------VISNDKYKSVLHRAVVNIDKERISIPTFYCPS    298
AtDLO2                          ----------------------------VISNEKYKSVLHRAVVNSDMERISIPTFYCPS    295
C.sativus_Cucsa.193360          ----------------------------VLSNDRYKSVLHRAVVNNATERISIPTFYCPS    299
C.papaya_evm.TU.sup_37.106      ----------------------------VISNDKYKSVVHRAVVNSERERISIPTFYCPS    296
E.grandis_v1_0.017927m.g        ----------------------------VISNDKYKSILHRAVVNCDRERISIPTFYCPS    298
E.grandis_v1_0.017897m.g        ----------------------------VISNDKYKSILHRAVVNCDRERISIPTFYCPS    298
E.grandis_v1_0.047536m.g        ----------------------------VISNDKYKSVLHRAVVNRNTERISIPTFCCPS    321
E.grandis_v1_0.017895m.g        ----------------------------VISNDKYKSVLHRAVVNCNTERISIPTFYCPS    298
E.grandis_v1_0.017876m.g        ----------------------------VISNDKYKSVLHRAVVNCNTERISIPTFYCPS    298
E.grandis_v1_0.015926m.g        ----------------------------VISNDKYKSVLHRAVVNCNTERISIPTFYCPS    298
VvDLO2                          ----------------------------VISNDRYKSVLHRAVVNCNKDRISIPTFYCPS    297
C.clementina_0.9_014263m.g      ----------------------------VLSNDRYKSVLHRAVVSRDKERISIPTFYCSS    302
P.persica_ppa008100m.g          ----------------------------VISNDKYKSVLHRAVVNCNSERISIPTFYCPS    296
P.persica_ppa008091m.g          ----------------------------VISNDKYKSVLHRAVVNCKSERISIPTFYCPS    296
R.communis_30068.t000102        ----------------------------VISNDRYKSVLHRAVVNSYEERISIPTFYCPS    295
M.esculenta_4.1_029834m.g       ----------------------------VISNNRYKSVLHRAVVNSDKERLSIPTFYCPS    296
                                                            .:**  * *  **. ..    *:*: :*
```

```
DMR6
B.distachyon_Bradi5g19240      NSAVIGPAAKLVGD---GDEPVYRSYTYDEYYNKFWSRN-LDQEHCLELFRGQK----   339
O.sativa_Os04g49194            NSAVISPARKLVAD---GDAPVYRSFTYDEYYKKFWSRN-LDQEHCLELFKGQ-----   340
O.sativa_Os10g39140            NSVELGPAKKLITD---DSPAVYRNYTYDEYYKKFWSRN-LDQEHCLELFRT------   342
S.bicolor_Sb01g030560          NHVVLGPAKKLVTE---DTPAVYRSYTYDEYYKKFWSRN-LDQEHCLELFRT------   336
ZmFNSI-1/ZmDMR6                NHVVLGPARKLVTE---DTPAVYRNYTYDKYYAKFWSRN-LDQEHCLELFRT------   336
B.distachyon_Bradi1g77040      NSVVLGPAEKLVGD---ASPAVYRNYTYDEYYKKFWSRN-LDQEHCLELFRT------   336
O.sativa_Os03g03034            NDVLIGPAQKLITD---GSPAVYRNYTYDEYYKKFWSRN-LDQEHCLELFRTTPTDTS   342
S.italica_Si036512m.g          NDVRIGPAAKLVGE---GAPAVYRDYTYAEYYGKFWSRN-LDQEHCLELFRT------   336
S.bicolor_Sb01g049030          NDVRIGPAAKLVTG---DTPAVYRDYTYAEYYAKFWSRN-LDQEHCLELFRT------   336
Z.mays_GRMZM2G475380           NDARIGPAARLLTD---GTPAVYRDYTYAEYYAKFWSRN-LDQEHCLELFRTPTS---   369
A.coerulea_v1.008143m.g        NDAIICPAV------KDGSPSMYKKFTYDEYYKKFWSGN-LDQQHCLELFKE------   335
E.salsugineum_TP2g22820        DCAVISPAKPLWEDEEDEAKPMYRDYTYAEYYKKFWSRN-LDQEHCLENFLNH-----   341
C.rubella_006g21660            DCAVMSPAKSLWEAEDSETKPIYRDFTYAEYYKKFWSRN-LDQEHCLENF--------   338
AtDMR6                         DCAVMSPAKPLWEAEDDETKPVYKDFTYAEYYKKFWSRN-LDQEHCLENFLNN-----   341
AlDMR6                         DCAVMSPAKPLWEAEDNETKPVYRDFTYAEYYKKFWSRN-LDQEHCLEYFRNN-----   341
C.sativus_Cucsa.273300         DDALITPAPLLSQ-----PSPIYRPFTYAQYYNTFWSRN-LDQQHCLELFKNHPP---   326
SlDMR6                         DSAKISAPKLLTED---GSPVIYQDFTYAEYYNKFWSRN-LDQQHCLELFKN------   337
M.guttatus_mgv1a009622m.g      DSANISAPKGLTGG---EDPAVYRDYTYNEYYKKFWSRN-LDQEHCLELFKN------   336
VvDMR6.1                       NDAVIGPAKPLTED---GSAPIYKNFTYAEYYKKFWGRD-LDQEHCLELFKN------   338
C.papaya_16416436              DDAVISPPKLLTAD---GSVAIYRDFTYAEYYKKFWSRN-LDQEHCLELFRNK-----   338
VvDMR6.2                       DDALISPARALTDE---GSAAIYRSFTYAEYYKKFWSRN-LDQEHCLEVFKN------   337
R.communis_29866.t000021       DDALISPAKPLTEG---ESGAVYRDFTYAEYYKKFWSRN-LDQEHCLELFKN------   337
M.truncatula_Medtr3g122530     NEALICPAKPLTED---GSGAVYRGFTYPEYYSKFWSRD-LEKEHCLEFFKNN-----   338
G.max_Glyma04g227900           DEALISPAKPLTEG---GSEAIYRGFTYAEYYKKFWSRN-LDQEHCLEFFKNK-----   338
G.max_Glyma06g137000           DEALISPAKPLTEH---GSEAVYRGFTYAEYYKKFWSRN-LDQEHCLELFKNK-----   338
G.max_Glyma06g14190            DEALISPAKPLTEH---GSEAVYRGFTYAEYYKKFWSRN-LDQEHCLELFKNK-----   338
E.grandis_v1_0.018520m.g       NDALISSPVSLLDN---GCGPTYRDFTYAEYYKKFWSRN-LDQEHCLELFKNQA----   339
E.grandis_v1_0.018494m.g       DDALISSPVSLLDN---GCGPTYRDFTYAEYYKKFWSRN-LDQEHCLELFKNQA----   339
E.grandis_v1_0.018496m.g       DDALISSPVSLLDN---GCGPTYRDFTYAEYYKKFWSRN-LDQEHCLELFKNQA----   339
P.persica_ppa008269m.g         DDALISPAKALTDD---GSAAIYRGYTYTEYYKKFWTRD-LNQEHCLELFKTDQ----   339
C.clementina_0.9_015118m.g     NDAMISPPKALTED---GSGAVYRDFTYAEYYSKFWSRN-LDQEHCLELFKN------   337
P.persica_ppa022381m.g         YDAVMEPAPQLVDD--H-HPPLYRSFTYAEFYEKFWDRG-LNTRSSLDLFQTTSHA--   345
P.persica_ppa019415m.g         YDAVMEPAPQLVDD--H-HPPLYRSFTYAEFYEKFWDRG-LNTRSSLDLFKTTSHA--   345
C.clementina_0.9_032642m.g     PDAVIAPAKDLIDER---HP-------------KFWNRG-LVDECCLDLFKASTA---   329
C.clementina_0.9_012078m.g     PDAVIAPAKDLIDER---HPAVYKNFTYAEYYQKFWNRG-L-DERCLDLFKASTA---   347
DLO
B.distachyon_Bradi5g19250      PDAVVAPAEALVDG--S-HPLAYRPFTYQEYYEEFWNMG-LESASCLDRFRPMD----   348
O.sativa_Os04g49210            PDAVIAPAGALVDGA-L-HPLAYRPFKYQAYYDEFWNMG-LQSASCLDRFRPNDQAV-   352
S.italica_Si010491m.g          PDAVIAPAGALVDD--A-HPLAYRPFTYQEYYDEFWNMG-LQSASCLDRFRPG-----   347
```

```
S.bicolor_Sb06g026350         PDGVIAPADALVDD--A-HPLAYRPFTYQEYYDEFWNMG-LQSASCLDRFRPGGSIE-   351
Z.mays_GRMZM2G050234          PDAVIAPADALVDD--G-HPLAYRPFTYQEYYDAFWNMG-LQSASCLDRFRPGGSLE-   351
G.max_Glyma03g42250           NDAIIGPAPQLIHHH-H-HPPQYNNFTYNEYYQNFWNRG-LSKETCLDIFKA------   350
G.max_Glyma16g01990           PDALIKPAPQLVDK--E-HPAQYTNFTYREYYDKFWIRG-LSKETCVDMFKAQD----   345
G.max_Glyma07g05420           PDALIKPAPKLVDN--E-HPAQYTNFTYREYYDKFWNRG-LSKETCVDMFKAQD----   345
VvDLO1 (G302E)                PDAVIGPAPGLVDH--G-HPALYRKFTYSEYFGKFWNRG-LATQSCLDMFKT------   344
R.communis_30076.t000026      PDAAIGPAPPLVDN--H-HPLLYTNFTYSQYYHKFWNRG-LATHTCLDMFKK------   344
AlDLO1                        TDAVIGPAHELINEQ-E-SLAVYRTFPFVEYWDKFWNRS-LATASCLDAFKASTT---   350
AtDLO1                        TDAVIGPAHELVNEQ-D-SLAIYRTYPFVEYWDKFWNRS-LATASCLDAFKAPTT---   349
AlDLO2                        EDAMIGPAQELINEE-EDSHAIYRNFTYAEYFEKFWDTA-FATESCIDSFKASTA---   351
AtDLO2                        EDAVISPAQELINEE-EDSPAIYRNFTYAEYFEKFWDTA-FDTESCIDSFKASTA---   348
C.sativus_Cucsa.193360        PEAMIGPAKELIHDE---HRPAFRNFTYSEYYQTFWSGE-LDTRRCLDLFRI------   347
C.papaya_evm.TU.sup_37.106    LDAVIGPAH---------RPSVYRNFSYGEYYSKFWSRSSLTAQACLDMFKA------   339
E.grandis_v1_0.017927m.g      PEALIGPAQGLIDHD---NPAVYRSFTYEEYYHKFWNRG-LRTECCLDMFKIPSA---   349
E.grandis_v1_0.017897m.g      PEALIGPAQGLIDHE---HPAVYRSFTYEEYYHKFWNRG-LRTECCLDMFKIPSA---   349
E.grandis_v1_0.047536m.g      PEGLIQPAQGLIDHE---HPPVYRSFTWEEFYQKFYHRG-LRTECCLDMFKIPSA---   372
E.grandis_v1_0.017895m.g      PEGLIRPAQGLIDHE---HPAVYKSFTYEEYYHKFWNRG-LRTECCLDMFKIHSA---   349
E.grandis_v1_0.017876m.g      PEGLIRPAQGLIDHE---HPAVYRSFTYEEYYHKFWNRG-LRTECCLDMFKIHSA---   349
E.grandis_v1_0.015926m.g      PEGLIRPAQGLIDHG---HPAVYRSFTYEEYYHKFWNRG-LRTECCLDMFKIPSA---   349
VvDLO2                        PDAVIGPSPELVDDD---HPAVYRNFTCEEYYTQFWNRG-LATESCLDTFKASTT---   348
C.clementina_0.9_014263m.g    PDAVIGPAKGLVDQD---HPAVYRDFTYAEYYKKFWNRG-LATECCLEMFKASSTV--   354
P.persica_ppa008100m.g        PDAVIGPAKDLISHD---QPAMYRNFTYAEYFEKFWNRG-LATECCLDLFKPN-----   345
P.persica_ppa008091m.g        PDAVIGPAKDLISHD---QPAMYRNFTYAEYFEKFWNRG-LATECCLDLFKPN-----   345
R.communis_30068.t000102      PDAVIGPAKDLIDPD---HPAAYREFTYAEYYEKFWDRG-LAKECCLDLFKTSTA---   346
M.esculenta_4.1_029834m.g     SDAVIGPAKDLIDND---HPAVYKHFTYAEYYEIFWNRG-LEKECCLDLFKISSA---   347
                                  :                             *:    :    .:: *
```
