# Supplementary material for: Mining Grapevine Downy Mildew Susceptibility Genes: A Resource for Genomics-Based Breeding and Tailored Gene Editing
Source: Biomolecules. 2021 Jan 28;11(2):181. doi: 10.3390/biom11020181 (PMC7912118; doi:10.3390/biom11020181)
Supplement: Supplementary file 1 [file biomolecules-11-00181-s001.zip › Supplementary_files_DEF/Figure_S1_legend_28.12.20.docx]

**Figure S1.** CLUSTALW alignment of bonafide and putative DMR6 and DLO proteins from different species. Amino acids important for the 2-DOG oxidase function (e.g.: the NYYPPCP stretch responsible for binding the 2-oxoglutarate substrate and the iron-binding HDH triplet) are highlighted in red. The DLO-DMR6 characterizing motif WRDY/FLRL is highlighted in yellow; R124 within the WRDY/FLRL motif, and R108 of the *Arabidopsis thaliana* DMR6-1 sequence were shown to be essential for the function and are as well highlighted in yellow [80]. Functional and applied aspects of the DOWNY MILDEW RESISTANT 1 and 6 genes in Arabidopsis. Utrecht University.). Amino acids of grapevine variants are highlighted in grey.

Bonafide DMR6 and DLO proteins are: *Zea mays* ZmFNSI-1/ZmDMR6, *A. thaliana* AtDMR6, AtDLO1 and AtDLO2; *A. lyrata* AlDMR6, AlDLO1 and AlDLO2; *Solanum lycopersicon* SlDMR6. The grapevine PN40024 DMR6 and DLO proteins (VvDMR6.1, VvDMR6.2, VvDLO1, and VvDLO2) are indicated in bold. The amino acid variants in the different grapevine accessions are indicated within parenthesis, and their position onto the PN40024 sequences is highlighted on a grey background.
